# Identification of Multiple Subsets of Ventral Interneurons and Differential Distribution along the Rostrocaudal Axis of the Developing Spinal Cord

**DOI:** 10.1371/journal.pone.0070325

**Published:** 2013-08-15

**Authors:** Cédric Francius, Audrey Harris, Vincent Rucchin, Timothy J. Hendricks, Floor J. Stam, Melissa Barber, Dorota Kurek, Frank G. Grosveld, Alessandra Pierani, Martyn Goulding, Frédéric Clotman

**Affiliations:** 1 Université catholique de Louvain, Institute of Neuroscience, Laboratory of Neural Differentiation, Brussels, Belgium; 2 Molecular Neurobiology Laboratory, The Salk Institute for Biological Studies, La Jolla, California, United States of America; 3 CNRS UMR 7592, Institut Jacques Monod, Université Paris Diderot, Sorbonne Paris Cité, Paris, France; 4 Erasmus MC Stem Cell Institute, Department of Cell Biology, Erasmus Medical Center, Rotterdam, The Netherlands; National University of Singapore, Singapore

## Abstract

The spinal cord contains neuronal circuits termed Central Pattern Generators (CPGs) that coordinate rhythmic motor activities. CPG circuits consist of motor neurons and multiple interneuron cell types, many of which are derived from four distinct cardinal classes of ventral interneurons, called V0, V1, V2 and V3. While significant progress has been made on elucidating the molecular and genetic mechanisms that control ventral interneuron differentiation, little is known about their distribution along the antero-posterior axis of the spinal cord and their diversification. Here, we report that V0, V1 and V2 interneurons exhibit distinct organizational patterns at brachial, thoracic and lumbar levels of the developing spinal cord. In addition, we demonstrate that each cardinal class of ventral interneurons can be subdivided into several subsets according to the combinatorial expression of different sets of transcription factors, and that these subsets are differentially distributed along the rostrocaudal axis of the spinal cord. This comprehensive molecular profiling of ventral interneurons provides an important resource for investigating neuronal diversification in the developing spinal cord and for understanding the contribution of specific interneuron subsets on CPG circuits and motor control.

## Introduction

Over the past two decades, an outline of molecular mechanisms that generate neuronal diversity in the developing spinal cord, coupled with a greater understanding of how these neurons are assembled into functional circuits, has begun to emerge. Repetitive motor activities, including swimming or walking, are controlled by complex central pattern generator (CPG) networks [1], [2], [3], [4]. Spinal CPG networks generate and coordinate the rhythmic and stereotyped patterns of motor activity independently of sensory or motor inputs [5], [6], [7]. These CPGs are broadly composed of motor neurons (MNs), the effectors of motor actions that are organized into functional motor pools, and several types of interneurons (INs) that serve to coordinate MN activity within and between CPG modules [2], [7], [8], [9], [10], [11], [12]. However, the topographic distribution of these different IN types along the rostrocaudal axis of the spinal cord remains poorly characterized, and the diversification of IN subsets that constitute the CPG has been only partially studied.

Among the neuronal populations that make up the spinal CPG circuitry, MNs are the component most well characterized (for review [13]). MN diversification along the rostrocaudal axis is regulated by extrinsic signals that include retinoic acid (RA), fibroblast growth factors (FGFs), Wnt proteins and GDF11 [14], [15], [16]. Combined activity of Hox transcription factors shapes the spinal cord along the rostrocaudal axis into a brachial, thoracic and lumbar regions [17], [18], [19], [20]. In each of these regions, MN diversify into distinct subclasses which form several columns according to a Hox code [15], [21], [22], [23]. At brachial levels, combined activities of Hoxc6 and Hoxc8 specify MNs into lateral motor column (LMC) [22], [24], [25] and medial motor column (MMC) neurons [26], [27]. By contrast, upon Hoxc9 activity at thoracic level, MN diversify into three columns called MMC, hypaxial motor column (HMC) and visceral preganglionic column (PGC) [21], [22], [24], [26], [27]. Similar to brachial level, Hoxd10 and Hoxc10 contribute to the specification of lumbar MNs into LMC and MMC neurons [22], [24], [25]. Interestingly, Hox cofactors such as Meis, Pbx or Foxp1 refine and constrain Hox activity within distinct MN subclasses. Foxp1, whose expression is selectively induced by Hox6/10 and Hoxc9 in LMC and PGC neurons, respectively, acts jointly with Hox transcription factors to specify LMC or PGC fate [21], [23], [27]. In addition, Foxp1 and Hox proteins synergistically control motor axon projections and axon targeting of LMC and of PGC neurons [21], [23]. Finally, Hox genes regulate the organization into specific motor pools [13], [24], [25].

Multiple distinct IN cell types are present in the adult spinal cord [12], [28], [29], [30], [31] (for review [32], [33]). Many of which are thought to arise from the V0, V1, V2 and V3 cardinal classes, although dorsal embryonic INs are also likely to contribute to the motor circuits (for review [9], [34], [35]). These ventral IN populations have been primarily characterized at brachial or at lumbar levels of the developing spinal cord [36], [37], and it is now apparent that these populations diversify into several subpopulations. Indeed, V0 INs subdivide into a dorsal (V0_D_), a ventral (V0_V_), a cholinergic (V0_C_) and a glutamatergic (V0_G_) complement [38]. V0_D_ are inhibitory commissural INs that control left/right alternation [39]. V1 INs sequentially differentiate into several inhibitory cell types including Renshaw cells (RCs), Ia INs and other unidentified subpopulations [40], [41], [42], [43], [44]. In contrast, V2 INs divide equally into V2a excitatory and V2b inhibitory INs [45], [46], [47], [48], [49], and in a small subpopulation of V2c INs [50]. While V2a INs regulate burst robustness and left/right coordination during walking, the physiological functions of V2b and V2c cells remain to be identified. Finally, a majority of V3 neurons are excitatory INs that segregate into a ventral (V3_V_) and a dorsal (V3_D_) subpopulation, which additionally differ in their electrophysiological properties [51], [52]. V3 neurons constitute a population of commissural INs that ensure a regular and balanced motor rhythm during walking by distributing excitatory drive towards both halves of the spinal cord [52].

Despite an increased understanding of the developmental programs that specify these cardinal ventral IN classes, very little is known about their topographic distribution and organization along the rostrocaudal axis of the spinal cord. The diversification of these cardinal classes of ventral INs into distinct subsets and the relationship between these subsets and adult IN populations also deserves further investigation. A major factor preventing progress is the incomplete characterization of molecular or genetic markers that define V0_D_, non-RCs V1 or V3_V_/V3_D_ subpopulations. While recent studies have described the expression of several transcriptional regulators in the developing spinal cord, including BhlhB5 [53] or Prdm8 [54], the Cut homeobox transcription factors of Onecut family such as HNF-6, OC-2 and OC-3 [55] and the bZIP transcription factors MafA, MafB [44] and cMaf, the relationship of these molecular markers to ventral IN subdivisions and known cell types has not been analyzed comprehensively.

Here, we characterized the topographic distribution of ventral INs along the rostrocaudal axis of the spinal cord using immunofluorescence labeling in combination with 3D imaging on whole mounted spinal cords. Our findings revealed pronounced differences in the distribution of ventral INs at brachial, thoracic and lumbar levels. We found that a majority of V0_D_ neurons contain BhlhB5 whereas Olig3 appears to define a majority of V3_V_ neurons. We also observed that each cardinal class of ventral INs can be subdivided into several subsets according to combinatorial expression of multiple transcription factors. Taken together, these findings provide evidence that the cardinal classes of ventral INs are differentially distributed along the rostrocaudal axis of the spinal cord and subdivide into different subsets characterized by distinct combinations of molecular markers.

## Materials and Methods

### Animals

All experiments were strictly performed respecting the European Community Council directive of 24 November 1986 (86-609/ECC) and the decree of 20 October 1987 (87-848/EEC). CD1 mice were raised in our animal facility and were treated according to the principles of laboratory animal care of the Animal Welfare Committee of the Université catholique de Louvain (Permit Number: UCL/MD/2009/008). The day of vaginal plug was considered as embryonic day (e)0.5 and embryos were collected at e12.5. Cohorts of a minimum of 3 embryos per experiment were used.

The *Dbx1* ^LacZ/+^, *En1* ^Cre/+^;ZnG, *Sim1* ^Cre/+^;*Rosa26* ^floxstop-tdTomato^ mutant mice were previously described [52], [56], [57]. The specificity controls of ß-galactosidase, Cre recombinase or GFP expression for each mouse line were provided in the cited references. *Gata3* ^Cre/+^;*Rosa26* ^floxstop-tdTomato^ mice were generated by breeding *Gata3* ^Cre/+^ mice (Zhang et al., submitted) with *Rosa26* ^floxstop-tdTomato^ mice. Characterization of *Gata3* ^Cre/+^;*Rosa26* ^floxstop-tdTomato^ mouse line was provided in the cited reference. In addition, our immunofluorescent analyses in the developing spinal cord indicated that Cre recombinase and tdTomato expression was restricted to the V2b and V2c interneurons (data not shown).

Mouse embryos were collected at e12.5 and decapitated before fixation. Embryos were fixed in PBS/4% PFA at 4°C for 25 minutes. They were washed thrice in cold PBS before incubation in PBS/30% sucrose overnight at 4°C. They were embedded in PBS/7.5% gelatin/15% sucrose before being frozen at −55°C. Embryos were cut at 14 µm in a Leica CM3050 cryostat and cryosections were stored at −20°C. For whole mount experiments, the whole spinal cord was removed, fixed in cold PBS/4% PFA for 25 minutes and were processed immediately.

### Immunofluorescent labelings

Cryosections or whole spinal cord were saturated with PBS/0.1% Triton/10% horse serum for 30 minutes and incubated with the primary antibodies diluted in the same solution at 4°C overnight or for 4 days, respectively. After 3 washes in PBS/0.1% Triton, the secondary antibodies, diluted in PBS/0.1% Triton/10% horse serum, were added for 30 minutes at room temperature. Slides or whole spinal cord were washed thrice in PBS/0.1% Triton before a final wash in PBS, and were mounted with Fluorescent mounting medium (DAKO).

Details of the sources of the primary or secondary antibodies, and their characterization by us or by others are indicated in Table 1 and Table 2, respectively. All secondary antibodies gave signals in the spinal cord only in the presence of corresponding primary antibodies, but not when applied on spinal cord sections alone.

**Table 1:** List of the primary antibodies used in this study.

Primary antibody used	Dilution	Supplier	Reference
Rabbit anti-Arx	1∶1000	Dr. Jamel Chelly	[124]
Chicken anti-b-galactosidase	1∶2000	Abcam	ab9361
Goat anti-BhlhB5	1∶1000	Santa Cruz	sc-6045
Rabbit anti-BhlhB5	1∶2000	Dr. Sarah E. Ross	[70]
Rat anti-BhlhB5	1∶2000	Dr. Sarah E. Ross	[70]
Mouse anti-Brn3a	1∶500	Santa Cruz	sc-8429
Guinea pig anti-Brn3a	1∶5000	Dr. Jane Johnson	[125]
Rabbit anti-Chx10	1∶5000	Dr. Kamal Sharma	[47]
Sheep anti-Chx10	1∶500	Exalpha Biologicals	X1179P
Guinea pig anti-Chx10	1∶3000	Dr. Sam Pfaff	[47]
Mouse anti-Calbindin	1∶10000	Swant	CB300
Rabbit anti-Calbindin	1∶10000	Swant	CB38
Mouse anti-Evx1	1∶2000	DSHB	99.1-3A2
Rabbit anti-Evx1	1∶300	Dr. Martyn Goulding	[65]
Rabbit anti-Foxd3	1∶5000	Dr. Thomas Müller	[81]
Guinea pig anti-Foxd3	1∶5000	Dr. Thomas Müller	[81]
Goat anti-Foxp1	1∶1000	R&D Systems	AF4534
Goat anti-Foxp2	1∶2000	Abcam	ab1307
Rabbit anti-Foxp4	1∶400	Genway Biotech	18-003-43528
Guinea pig anti-Gata2	1∶3000	Dr. Kamal Sharma	[47]
Mouse anti-Gata3	1∶400	Santa Cruz	sc-268
Rat anti-Gata3	1∶20	Dr. Frank Grosveld	[50]
Chicken anti-GFP	1∶2000	Abcam	ab290
Rabbit anti-GFP	1∶2000	Aves Labs	GFP-1020
Guinea pig anti-HNF-6	1∶6000	Our laboratory	[126]
Rabbit anti-HNF-6	1∶50	Santa Cruz	sc-13050
Rabbit anti-Lbx1	1∶5000	Dr. Thomas Müller	[127]
Guinea pig anti-Lbx1	1∶5000	Dr. Thomas Müller	[127]
Guinea pig anti-MafA	1∶10000	Dr. Thomas Müller	[128]
Rabbit anti-MafA	1∶500	Novus Biological	NB400-137
Rabbit anti-MafB	1∶5000	Dr. Hagen Wende	[128]
Rabbit anti-cMaf	1∶5000	Dr. Hagen Wende	[129]
Mouse anti-Nkx2.2	1∶20	DSHB	74.5A5
Rat anti-Nurr1	1∶2000	Dr. Yuichi Ono	[130]
Rat anti-OC-2	1∶400	Our laboratory	[131]
Guinea pig anti-OC-3	1∶6000	Our laboratory	[132]
Rabbit anti-Olig3	1∶5000	Dr. Thomas Müller	[81]
Guinea pig anti-Olig3	1∶5000	Dr. Thomas Müller	[81]
Rabbit anti-Pax2	1∶100	Covance	PRB-276P
Goat anti-Pax6	1∶500	Santa Cruz	sc-7750
Rabbit anti-Pax6	1∶2000	Covance	PRB-278P
Mouse anti-Pax6	1∶1000	DSHB	PAX6
Rabbit anti-Pitx2	1∶5000	Capra Science	PA 1020-100
Rabbit anti-Prdm8	1∶200	Pr. Yoichi Shinkai	[54]
Mouse anti-Prdm8	1∶200	Pr. Yoichi Shinkai	[54]
Guinea pig anti-Prdm8	1∶2000	Dr. Sarah E. Ross	[70]
Rabbit anti-Prdm8	1∶2000	Dr. Sarah E. Ross	[70]
Rabbit anti-Prox1	1∶1000	Covance	PRB-238C
Goat anti-Prox1	1∶500	R&D Systems	AF2727
Rabbit anti-RFP	1∶2000	Rockland Inc.	600-401-379
Goat anti-Sox1	1∶500	Santa Cruz	sc-17318
Rabbit anti-Sox1	1∶400	Dr. Stavros Malas	[50]
Guinea pig anti-Sox1	1∶400	Dr. Stavros Malas	[50]
Rabbit anti-SCIP (Pou3f1)	1∶500	Dr. Dies Meijer	[133]

**Table 2:** List of the secondary antibodies used in this study.

Secondary antibody used	Dilution	Supplier	Reference
Donkey anti-mouse/AlexaFluor488	1∶2000	Life Science	A21202
Donkey anti-mouse/AlexaFluor594	1∶2000	Life Science	A21203
Donkey anti-mouse/AlexaFluor647	1∶2000	Life Science	A31571
Donkey anti-rabbit/AlexaFluor647	1∶2000	Life Science	A31573
Donkey anti-rat/AlexaFluor594	1∶2000	Life Science	A21209
Donkey anti-goat/AlexaFluor488	1∶2000	Life Science	A11055
Donkey anti-sheep/AlexaFluor594	1∶2000	Life Science	A11016
Goat anti-guinea pig/AlexaFluor594	1∶2000	Life Science	A11076
Goat anti-mouse IgG_1_/AlexaFluor594	1∶2000	Life Science	A21125
Goat anti-mouse IgG_2a_/AlexaFluor488	1∶2000	Life Science	A21131
Donkey anti-chicken/Dylight488	1∶1000	Jackson ImmunoResearch	703-485-155
Donkey anti-chicken/Dylight594	1∶1000	Jackson ImmunoResearch	703-515-155
Donkey anti-mouse/AlexaFluor647	1∶1000	Jackson ImmunoResearch	715-605-151
Donkey anti-rat/AlexaFluor647	1∶1000	Jackson ImmunoResearch	712-605-153
Donkey anti-sheep/AlexaFluor594	1∶1000	Jackson ImmunoResearch	713-585-147
Donkey anti-sheep/AlexaFluor647	1∶1000	Jackson ImmunoResearch	713-605-147
Donkey anti-rabbit/AlexaFluor488	1∶1000	Jackson ImmunoResearch	711-545-152
Donkey anti-rabbit/AlexaFluor594	1∶1000	Jackson ImmunoResearch	711-585-152
Donkey anti-guinea pig/AlexaFluor488	1∶1000	Jackson ImmunoResearch	706-545-148
Donkey anti-guinea pig/AlexaFluor647	1∶1000	Jackson ImmunoResearch	706-605-148

### Imaging and quantitative analyses

Immunofluorescence images of cryosections were acquired on a Zeiss Axio Cell Observer Z1 confocal microscope with the Zeiss AxioVision Rel. 4.8 software and processed with Adobe Photoshop CS5 software.

Quantifications were performed on red or green or blue layer of acquired confocal images and double or triple labeling cells were processed by subtractive method [55]. For each e12.5 embryo (n = 3), labeled cells were quantified using the count analysis tool of Adobe Photoshop CS5 software. Neurons that were positive for molecular markers described above were counted unilaterally on three sections at three different levels of the brachial, thoracic and lumbar regions. Depending on the rostrocaudal level, the amount of cells that was counted for each IN population was: 91–219 cells for V0, 169–257 cells for V1, 50–107 cells for V2a, 59–103 cells for V2b and 41–76 cells for V3. Raw data were exported from Adobe Photoshop CS5 software to SigmaPlot v12 software and processed in order to generate histogram figures. Analyses of the Variance performed for each experiment by One Way ANOVA test indicated a low and non-significant variability from mouse to mouse (p = 0,169 to 0,757). Each histogram generated in this study was normalized to the total amount of cells in each ventral interneuron population that was set at 100.

Z-stack images of whole mount labelings in open-book spinal cord preparations were obtained using LSM510 and Zeiss Axio Cell Observer Z1 confocal microscope. Three-dimensional (3D) image reconstructions were made and processed using Zeiss ZEN 2011 black edition (release version 7.0.0.285) software. Optical sections in Z-series collections were collected at 0.540 µm intervals (objective: LD LCI Plan-Apochromat 25×0.8 Imm Korr DIC M27, camera: AxioCamMR3). Brightness and contrast were modified using Zeiss ZEN 2011 software, then movies were generated with Zeiss ZEN 2011 software.

## Results

### Ventral spinal INs are differentially distributed along the rostrocaudal axis of the spinal cord

**Figure 1:**
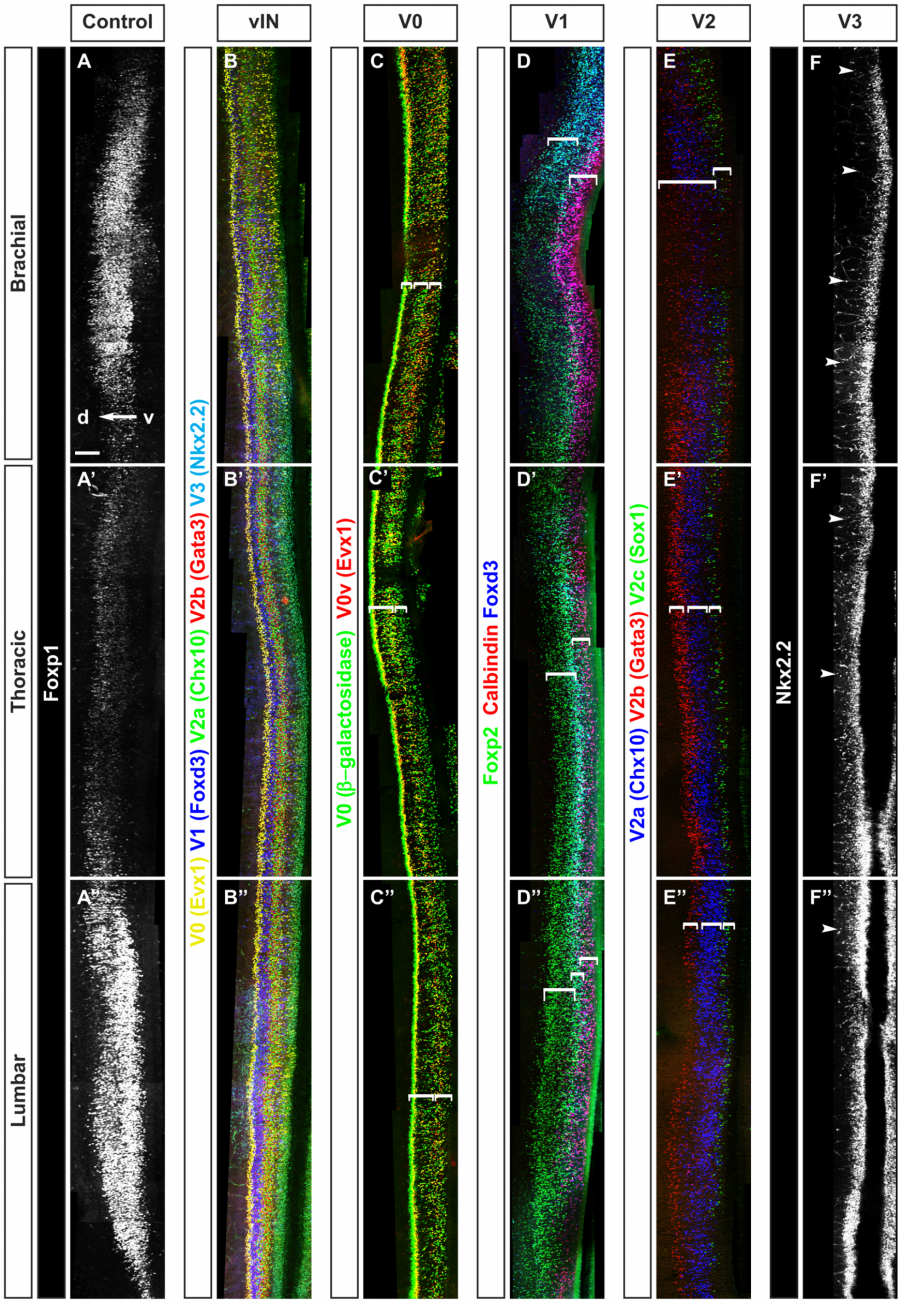
Ventral spinal interneurons are differentially distributed along the rostrocaudal axis of the spinal cord. Whole mount immunofluorescence labeling of Foxp1 or ventral interneuron markers on whole mount spinal cord of mouse embryo at e12.5. (A–A″) Foxp1 (white) shown as reference to delineate the brachial, thoracic and lumbar regions of the developing spinal cord. (B–B″) Differential distribution of ventral spinal interneurons including V0_V_ (yellow), V1 (blue), V2a (green), V2b (red) and V3 (cyan) at brachial (B), thoracic (B′) or lumbar (B″) levels of the spinal cord. (C–C″) V0 interneurons including V0_V_ (red or yellow) and V0_D_ (green) display distinct distribution pattern at brachial (C), thoracic (C′) or lumbar (C″) levels in Dbx1^LacZ/+^ embryos. (D–D″) V1 interneurons including Renshaw cells (red or magenta) and V1 non-Renshaw cells that contain Foxp2 (green) or Foxd3 (blue) are differentially distributed at brachial (D), thoracic (D′) or lumbar (D″) along the rostrocaudal axis. (E–E″) V2 interneurons including V2a (blue), V2b (red) and V2c (green) exhibit distinct distribution pattern at brachial (E), thoracic (E′) or lumbar (E″) levels. (F–F″) By contrast, V3 interneurons (white) display relative homogenous distribution, along the rostrocaudal axis of the spinal cord. The white brackets indicate distinct columnar organization within V0, V1 and V2 neurons while white arrowheads show few cells distributed outside V3 neuron column. The white arrow indicates the ventral (v) to dorsal (d) axis. Scale bar = 100 µm.

Different classes of cardinal INs have been described in the developing ventral spinal cord [36], [37], [58], [59], [60]. However, most of these populations have been characterized at a single rostrocaudal level, and the respective distribution of these embryonic neuronal populations along the rostrocaudal axis of the spinal cord has not been extensively described. To address this question, we first examined the topographic distribution of the ventral INs at e12.5 by immunofluorescence on whole mount spinal cord combined with 3D imaging. At this developmental stage, the ventral spinal INs have been generated and a majority of them have reached their final position. Furthermore, the ventral spinal cord is still devoid of glial cells [61], [62], [63] and ventral INs have not yet undergone developmental cell death [64], which, at a later stage, would complicate the analyses. Multiple labeling of each cardinal interneuron population revealed that these cells are not identically distributed along the rostrocaudal axis of the spinal cord (Fig. 1B–B″). Indeed, at thoracic levels, a ventral to dorsal (Fig. 1A) series of columns corresponding to V3, sparse V1, intermingled V2a, V2b and V3 and finally V0_V_ INs were observed (Fig. 1B′). At variance, the organization, the size and the relative position of these columns seemed different at brachial compared to lumbar levels (Fig. 1B–B″). To gain further insights into these possible differential distributions, each cardinal class of ventral INs was analyzed separately.

The V0 INs were visualized in Dbx1^LacZ/+^ mouse embryos, in which ß-galactosidase is detected in V0 progenitors and in a majority of differentiating V0 cells [56], but not in V1 or in dorsal dI6 INs (Fig. S5A–F″). Evx1 was detected as a specific marker of V0v INs [56], [65]. At brachial levels, the V0 neurons were organized into 3 columns (brackets in Fig. 1C, Fig. S1A, and Movie S1 in Movie Collection S1). The dorsal column corresponded to the V0 progenitors and newly-born V0_V_ (Evx1^+^) or V0_D_ neurons. The intermediate column contained a large group of V0_D_ intermingled with some V0_V_ cells. The ventral column was composed of a large group of V0_V_ intermingled with fewer V0_D_ INs. By contrast, at thoracic levels, the intermediate column included a large portion of V0_V_ and fewer V0_D_, while the ventral column was composed almost exclusively of V0_D_ cells (brackets in Fig. 1C′, Fig. S1B, Movie S2 in Movie Collection S1). At lumbar levels, we observed two major columns distributed between a dorsal complement of tightly packed V0 progenitors and newly-born V0_D_ or V0_V_ cells, and a ventral complement with intermingled V0_D_ and V0_V_ neurons (brackets in Fig. 1C″, Fig. S1C, Movie S3 in Movie Collection S1). Note that a small fraction of V0 INs displayed low levels of ß-galactosidase at brachial levels, probably due to ß-galactosidase degradation in the earliest-born cells. Furthermore, the distribution of V0 INs (and of the other cardinal populations, see below) at brachial or lumbar levels varied according to the differential distribution of the MNs in these regions, as previously reported [41]. Thus, V0 INs exhibited a differential distribution of the V0_D_ and V0_V_ subpopulations, including changes in their relative dorso-ventral position, along the rostrocaudal axis of the spinal cord.

V1 INs divide into RCs (Foxd3^+^/Calbindin^+^) and V1 non-RC INs (Foxp2^+^/Foxd3^+^ or Foxd3^+^) that include inhibitory Ia INs and other uncharacterized populations [40], [42], [44], [66]. At brachial levels, V1 neurons were distributed in a ventral column that contained only RCs and a large dorsal column composed of numerous Foxp2^+^ V1 neurons and rare Foxd3^+^ V1 cells (brackets in Fig. 1D, Fig. S2A, and Movie S1 in Movie Collection S2). At thoracic levels, the V1 gathered in a less-extended dorso-ventral portion of the spinal cord (brackets in Fig. 1D′) with a progressive reduction in RCs from rostral to caudal, and Foxp2^+^/Foxd3^+^ cells grouped in a central column (brackets in Fig. 1D′, Fig. S2B, Movie S2 in Movie Collection S2). At lumbar levels, V1 INs were slightly more numerous than at thoracic levels. However, fewer RCs were observed and were spread more dorsally while Foxd3 levels decreased along the cranial to caudal axis (Fig. 1D″, Fig. S2C, Movie S3 in Movie Collection S2). Thus, the amount and the divisions of V1 neurons varied between limb and thoracic levels.

V2 INs segregate into V2a (Chx10^+^), V2b (Gata3^+^) and V2c (Sox1^+^) subpopulations [47], [50], [67]. At brachial levels, V2b neurons were widely distributed from very ventral to medial regions, whereas V2a intermingled with V2b neurons in a central column and V2c gathered close to the ventral most V2b cells (brackets in Fig. 1E, Fig. S3A, and Movie S1 in Movie Collection S3). At thoracic levels, V2b INs were more dorsally restricted, being almost absent from ventral regions, whereas V2a gathered in a narrower central column directly ventral to the V2b cells (brackets in Fig. 1E′, Fig. S3B, Movie S2 in Movie Collection S3). At lumbar levels, V2a INs were more numerous and expanded along the dorso-ventral axis. V2b cells were less numerous in the rostral portion but more numerous and more extended along the dorso-ventral axis in the caudal portion of the lumbar region. V2c were less numerous with respect to V2a and V2b (brackets in Fig. 1E″, Fig. S3C, Movie S3 in Movie Collection S3). Thus, the V2 INs presented a differential distribution along the rostrocaudal axis of the spinal cord.

Finally, V3 INs labeled for their specific marker Nkx2.2 were organized in a single ventral column homogenous along the rostrocaudal axis of the spinal cord, although a few of them were distributed dorsally more frequently within the brachial and thoracic levels, but less frequently in the lumbar region (arrowheads in Fig. 1F–F″, arrowheads in Fig. S4A–C, Movies S1, S2, S3 in Movie Collection S4).

Together, these observations provided evidence that the cardinal classes of ventral INs are differentially distributed along the rostrocaudal axis of the spinal cord except for V3, which exhibited a more homogenous distribution pattern.

### Ventral spinal INs can be subdivided into subsets with distinct molecular identity

The four cardinal classes of ventral INs give rise to dozens of distinct neuronal populations in the adult, suggesting that they subdivide into numerous subsets. Differential location of these subsets may contribute to the differential distribution reported above. In an effort to address this question and to identify molecular markers of subsets of embryonic ventral INs, we analyzed the distribution of a set of transcription factors that have been described in the ventral part of the developing spinal cord relative to established markers of ventral IN populations. The transcription factor set included BhlhB5 (also known as Bhlhe22) [53], [68], [69], Prdm8 [54], [70], HNF-6, OC-2, OC-3 [55], Nurr1 [71], Pax2 [72], Pax6, MafA, MafB or cMaf [73], Pitx2 [38], Olig3 [74], Pou3F1 and Pou4F1 [75].

### Homogenous subsets of V0 INs along the rostrocaudal axis of the spinal cord

**Figure 2:**
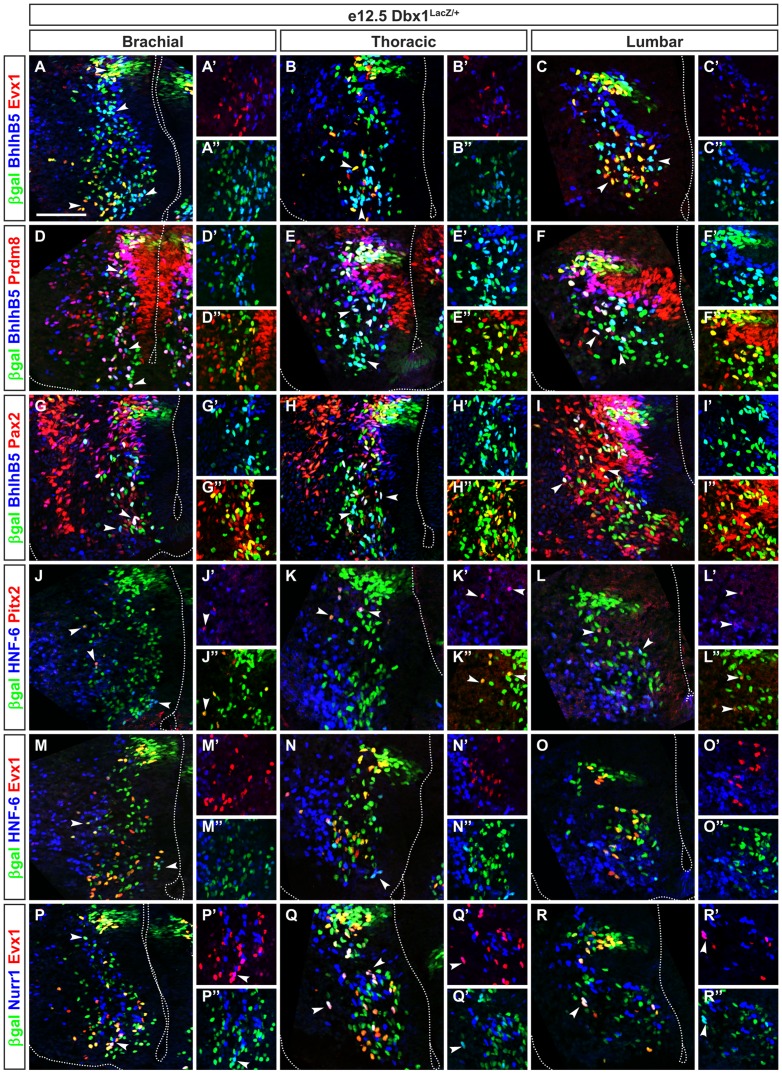
Homogenous distribution of V0 interneuron subsets along the rostrocaudal axis of the spinal cord. (A–A″) Immunofluorescence labeling on transverse section at brachial levels of the spinal cord of Dbx1^LacZ/+^ embryo at e12.5. V0_D_ interneurons contain BhlhB5 (cyan) are intermingled with Evx1^+^ V0_V_ neurons (yellow or red). Most of V0_V_ cells are located ventrally as indicated by left arrowhead while V0_D_ are homogenously distributed between the vicinity of the V0 progenitor domain and the ventro-medial region of the spinal cord as shown by right arrowhead. (B–B″) At thoracic levels, V0_V_ (yellow or red) amounted less in the ventral territory of V0 interneurons compared to brachial levels (see arrowhead). Conversely, most of V0_D_ in magenta are gathered in this ventral territory as indicated by arrowhead. (C–C″) At lumbar levels, V0_V_ (yellow or red) form a relatively homogenous group located in the ventral territory (left arrowhead), while V0_D_ (cyan) distribute among dorsal, ventro-medial and ventro-lateral territories (right arrowhead). (D–D″) At brachial levels, Prdm8 is detected V0 progenitors and in subsets of V0_V_ (yellow) located close to the central canal (arrowheads). By contrast, Prdm8 is present in a few number of V0_D_ cells (white) as shown by bottom arrowhead. (E–E″) At thoracic levels, Prdm8^+^ V0_V_ interneurons (yellow) are located close to progenitor domain and in a dorso-ventral direction, while the amount of Prdm8^+^ V0_D_ (white) is increased (see arrowheads). (F–F″) At lumbar levels, subsets of V0_D_ that are Prdm8^+^ (white) are located in a dorso-ventral direction between the V0 progenitor domain and V0_V_ neurons (yellow or green) as indicated by arrowheads. (G–G″) At brachial levels, Pax2 is present in a large subset of V0_D_ neurons (white) and in a small subset of V0_V_ neurons (yellow) distributed between the V0 progenitor domain and V0_V_ ventral territory (see arrowheads). (H–I″) The Pax2^+^ V0 subsets are similarly distributed at thoracic and lumbar levels. (J–J″) At brachial levels, Pitx2 is expressed in V0_C_ interneurons (yellow) which are located laterally to the V0 interneuron migration flux as indicated by arrowheads. A portion of V0_C_ that co-expresses HNF-6 (magenta) is present ventrally (see bottom arrowhead). (K–K″) At thoracic levels, most of V0_C_ express HNF-6 (magenta). V0_C_ are detected laterally to the V0 progenitor domain and dorsally to V0_V_ ventral territory as indicated by arrowheads. (L–L″) At lumbar levels, the amount of V0_C_ (yellow) is reduced compared to brachial or thoracic levels (see left arrowhead). (M–M″) At brachial levels, HNF-6 is detected in a small subset of Evx1^+^ V0_V_ interneurons (white or magenta) as indicated by left arrowhead and in a subset of V0_D_ (cyan) indicated by right arrowhead. (N–N″) At thoracic levels, HNF-6 is restricted to a few number of V0_V_ (white) and of V0_D_ (cyan) indicated by arrowhead. (O–O″) At lumbar levels, HNF-6 is present in very few Evx1^+^ cells (white or magenta) or V0_D_ (cyan) neurons compared to brachial or thoracic regions. (P–P″) At brachial levels, Nurr1 expression is detected in a small subset of V0 including V0_V_ (white or magenta) indicated by bottom arrowhead and V0_D_ (cyan) located ventrally, close to the floor plate (see arrowhead in insets). (Q–Q″) At thoracic levels, Nurr1^+^ subsets of V0_V_ (white or magenta) indicated by arrowheads or of V0_D_ (cyan) are expanded. (R–R″) By contrast, at lumbar levels, Nurr1 expression is only detected in small subsets within V0_V_ (white or magenta) indicated by arrowhead and V0_D_ (cyan). Scale bar = 100 µm.

Previous studies established that V0 INs diversify into distinct subpopulations including V0_D_, V0_V_ (Evx1^+^), V0_C_ (Evx1^+^/Pitx2^+^/ACh^+^) and V0_G_ (Evx1^+^/Pitx2^+^) neurons [38], [39], [56]. Using Dbx1^LacZ/+^ embryos, we determined whether additional candidate markers were present in V0 INs. Surprisingly, we found that BhlhB5, a transcriptional repressor expressed in dorsal dI6 INs, V1 and V2a INs [53], [68], [69] was additionally detected in 77% to 89% of Evx1^−^ V0 INs, namely V0_D_ (Fig. 2A–C″; Fig. S5A–D″; Fig. 7A–C). In contrast, BhlhB5 was not present in Evx1^+^ V0_V_ INs (Fig. 2A–C″), as described previously [53]. Hence, BhlhB5 is a marker for a majority of V0_D_ neurons. This observation also highlights further diversification of V0_D_ INs into BhlhB5^+^ or BhlhB5^−^ cells.

At brachial levels, the BhlhB5^+^ V0_D_ INs accounted for 50% of the V0 INs (Fig. 7A) and were located close to the central canal. About half of these cells contained the PR Domain Zinc Finger Protein Prdm8, a cofactor of BhlhB5 (Fig. 2D–D″, Fig. 7B) or Pax2 (Fig. 2G–G″, Fig. 7C). Smaller subsets were labeled for Onecut factors (arrowheads in Fig. 2J–J″, M–M″; Fig. 7D), Pax6 or the orphan nuclear receptor Nurr1 (Nr4a2), a protein previously detected in glutamatergic cortical neurons and dopaminergic neurons (arrowheads in Fig. 2P–P″; Fig. 7A, E; data not shown) [76], [77]. V0_V_ accounted for 40% of the V0 INs (Fig. 7A). They migrated ventro-medially to become located lateral to BhlhB5^+^ V0_D_ INs (Fig. 2A–A″). They divided into major subsets labeled for OC-2 or OC-3 (Fig. S6A–B) and Nurr1 (Fig. 2P–P″, Fig. 7E) and into minor subsets containing Prdm8^+^ (Fig. 2D–D″, Fig. 7B), HNF-6^+^ (Fig. 2J–J″, Fig. 7D) and Pax2^+^ (Fig. 2M–M″, Fig. 7C). Finally, V0_C_ and V0_G_ INs defined by Pitx2 formed a small subset of V0_V_ (Fig. 2G–G″) and a majority of them contained HNF-6 (arrowheads in Fig. 2J–J″, Fig. 7D). At thoracic and lumbar levels, the proportion of V0_D_ and V0_V_ did not significantly change compared to brachial levels (Fig. 2B–B″, Fig. 7A). Likewise, the proportions of V0 subsets remained relatively stable, with the exception of an increase in HNF-6^+^ and in OC-3^+^ V0_V_ that correlated with a decrease in HNF-6^+^ and in OC-3^+^ V0_D_ INs at thoracic levels of the spinal cord (Fig. 7A–E, Fig. S6A–B).

Thus V0_V_ and V0_D_ proportion did not significantly change along the rostrocaudal axis of the spinal cord, and each V0 subpopulation can be divided into several subsets according to a combinatorial code that includes Pax2, Prdm8, Pax6, Nurr1 or Onecut factors, which are differentially distributed between brachial, thoracic and lumbar levels.

### V1 interneuron subsets are differentially distributed along the rostrocaudal axis of the developing spinal cord

V1 INs characterized by Foxd3 and En1 expression arise from the p1 progenitor domain defined by combined expression of Dbx2, Nkx6.2 and Pax6 [56], [57], [78], [79]. Neurons of this class rapidly diversify into several inhibitory interneuron subpopulations including RCs characterized by the presence of calbindin and Foxd3, and V1 non-RCs containing Ia INs and other V1 subpopulations characterized by Foxp2, Foxd3, and En1 [40], [43], [44], [66], [79]. Noticeably, Foxd3 and Foxp1 are also present in dI2 INs, but these cells are not yet located in the ventral spinal cord at e12.5 [80], [81].

**Figure 3:**
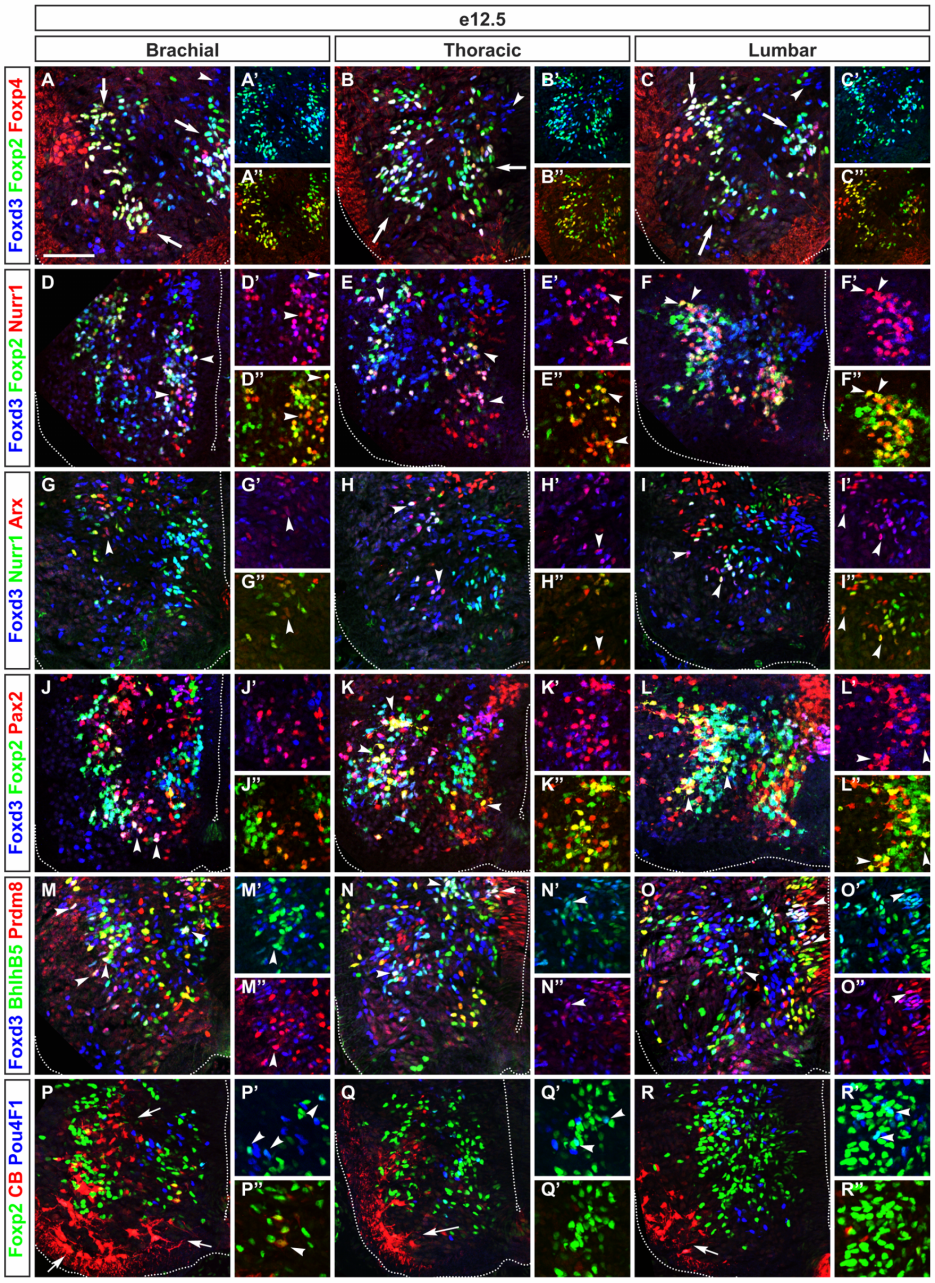
Differential distribution of V1 interneuron subdivisions along the rostrocaudal axis of the spinal cord. (A–A″) Immunofluorescence labeling on transverse section at brachial levels of embryonic spinal cord at e12.5 shows that V1 interneurons are subdivided into 3 large subsets (arrows) containing Foxp2 (cyan). Foxp4 is detected in a portion of these Foxp2^+^ V1 neurons (white). (B–B″) At thoracic levels, Foxd3^+^ V1 neurons form 2 groups (arrows) that contain Foxp2 (in green/cyan) and Foxp4 (in red/white). (C–C″) At lumbar levels, V1 interneurons distribute among 3 groups (arrows) and the ratio between Foxp2^+^ neurons and Foxp4^+^/Foxd3^+^ cells decreased compared to brachial or thoracic levels. (D–D″) At brachial levels, Nurr1 is detected in subsets (arrowheads) within 2 large V1 groups containing Foxp2 (yellow). (E–E″) At thoracic levels, Nurr1^+^ subsets (white) are intermingled with Foxp2^+^ group (cyan and green) close to the central canal and/or (in/with Foxp2^+^ group close) to motor neurons as indicated by arrowheads. (F–F″) At lumbar levels, Nurr1^+^ subsets are included in 2 groups of V1 that contain Foxp2 (yellow) indicated by arrowheads or Foxd3 and Foxp2 (white). (G–G″) At brachial levels, Arx is present in a few number of Foxd3^+^ V1 neurons (magenta) as indicated by arrowhead. (H–I″) By contrast, at thoracic and lumbar levels, Arx is observed in subsets of Foxd3^+^ (magenta) and of Foxd3^+^/Nurr1^+^ (white) V1 neurons (arrowheads). (J–J″) At brachial levels, Pax2 expression defines subsets in Foxd3^+^ (magenta) and in Foxp2^+^/Foxd3^+^ (white) V1 interneurons (arrowheads). (K–K″) At thoracic levels, Pax2 is distributed in Foxd3^+^ (magenta), Foxd3^+^/Foxp2^+^ (white) and Foxp2^+^ subsets (yellow) as indicated by arrowheads. (L–L″) At lumbar levels, Pax2^+^ subsets (yellow or white) are located within V1 containing Foxp2 located medially or close to motor neurons (arrowheads). (M–M″) At brachial levels, BhlhB5 is detected in subsets of Foxd3^+^ V1 neurons (cyan) located close to the central canal or to motor neurons. Prdm8 is present in small subsets of V1 neurons located close to V1 progenitor domain or more ventrally, close to motor neurons. A small subset of V1 that co-express BhlhB5 and Prdm8 (white) is observed in the vicinity of the motor neurons (arrowheads). (N–N″) At thoracic levels, the amount of V1 neurons that contain BhlhB5 (cyan), or BhlhB5 and Prdm8 (white) indicated by arrowheads, is increased while a few number of Prdm8^+^ V1 cells is detected. (O–O″) At lumbar levels, the amount of cells corresponding to Prdm8^+^ (magenta) or BhlhB5^+^ V1 neurons (cyan) decreases while BhlhB5^+^/Prdm8^+^ co-expressing V1 (white) indicated by arrowheads, increase compared to the thoracic region. (P–P″) At brachial levels, Pou4F1 is present in a few Foxp2^+^ V1 neurons (cyan) indicated by arrowheads. Renshaw cells (red) are distributed in 3 groups (arrows) with very few containing Foxp2 (yellow) indicated by arrowhead. (Q–Q″) At thoracic levels, the amount of Renshaw cells (red) decreases (arrow) while Foxp2^+^ V1 expressing Pou4F1 (cyan) slightly increases (arrowheads). (R–R″) At lumbar levels, similar to thoracic levels, the amount of Renshaw cells (red) decreases (arrow) but Pou4F1^+^ subset (cyan) is detected dorsally, close to motor neurons (arrowheads). Scale bar = 100 µm.

At brachial levels, non-RC V1 amounted for 70% of the V1 INs. A large non-RC subset contained Foxp2, partly in combination with Foxp4 and/or Foxd3 (Fig. 3A–A″, Fig. 7F). This large subset spatially divided into 3 groups, i.e. a medial, a ventro-lateral and a lateral subdivision (arrows in Fig. 3A–A″). In addition, we detected a subset of non-RC V1 that contained Foxd3 but not Foxp2 nor Foxp4 in the vicinity of the p1 progenitor domain (arrowhead in Fig. 3A–A″, Fig. 7F). The Foxp2^+^ V1 subset could be further subdivided based on the distribution of Nurr1. This protein was detected in a small subset of V1 that contained Foxp2 but not Foxd3 located in the dorsal part of the lateral subdivision of V1 and in a large subset that contained Foxp2 and Foxd3 located in the medial subdivision (arrowheads in Fig. 3D–D″, Fig. 7G). Similarly, Pax2 was detected in the ventro-lateral subdivision that contained Foxp2 and Foxd3 (arrowheads in Fig. 3J–J″, Fig. 7H). At variance, Foxp1 was present in the medial subdivision of non-RC V1 INs and in the lateral subdivision that contained Foxd3, close to MNs (arrowheads in Fig. S5G–I″, Fig. 7J). Arx was detected in a small subset of the lateral group of Foxp2^+^/Nurr1^+^ V1 INs (arrowheads in Fig. 3G–G″, Fig. S6C), BhlhB5 and Prdm8 were partly codetected in a subset of V1 INs located close to the p1 domain that corresponds to newly-born V1 INs and in the lateral complement, close to the MNs (arrowheads in Fig. 3M–M″, Fig. S6D). Finally, Pou3F1 (also known as Scip or Oct6) and Pou4F1 (also known as Brn3a) were detected in a very limited number of V1 INs (arrowheads in Fig. 3P–P″, Fig. 7I–J, Fig. S5G–G″). On the other hand, RCs amounted for 30% of V1 cells (Fig. 7I). They divided into 3 distinct groups, i.e. a ventro-medial subdivision close to the lumen of the spinal cord, a ventro-lateral subdivision that bordered the MNs and a third group located dorsally to this ventro-lateral subdivision (arrows in Fig. 3P–P″). Foxp2 was detected in a few of these cells (arrowhead in Fig. 3P″, Fig. 7I).

At thoracic and lumbar levels, Foxp2^+^ non-RC V1 INs organized in a lateral and a medial subdivision (arrows in Fig. 3B–B″, Fig. 7F). Among these, the respective amount of each subset was relatively stable (Fig. 7F–J). However, V1 neurons containing exclusively Foxd3 were more numerous at thoracic levels whereas those containing exclusively Foxp2 decreased at lumbar levels, and V1 subsets characterized by the presence of Arx or Nurr1 were more abundant at thoracic or at lumbar levels, respectively (arrowheads in Fig. 3D–I″, Fig. 7G, Fig. S6C). In contrast, the Pax2^+^ subset was decreased at thoracic levels (Fig. 3J–L″, Fig. 7H), whereas the Bhlhb5^+^ one was increased (Fig. 3M–O″, Fig. S6D). RCs were progressively less numerous in more caudal regions, as reported previously [43], [44], [82], [83]. In addition, they gathered in a unique group located laterally, close to the basal lamina and the MNs (Fig. 3Q–Q″, Fig. 7I).

In summary, V1 INs were distributed into RCs and into 3 or 2 large subpopulations of Foxp2^+^ or Foxd3^+^ cells. Each V1 subpopulation except for RCs can be subdivided in several subsets distinguished by the single or the combinatorial expression of Pax2, Nurr1, Pou4F1, BhlhB5 or Prdm8, the distribution and the amount of which differ according to the rostrocaudal level.

### V2 interneuron subsets are homogeneous along the rostrocaudal axis of the spinal cord

V2 INs are produced from the p2 domain defined by combined expression of Foxn4, Mash1 (rename Ascl1), Lhx3 and Gata2 [47], [84], [85]. They are distributed between the excitatory V2a subpopulation defined by Chx10 expression and the V2b and V2c inhibitory subpopulations characterized by the presence of Gata2/Gata3 and Sox1, respectively [50].

**Figure 4:**
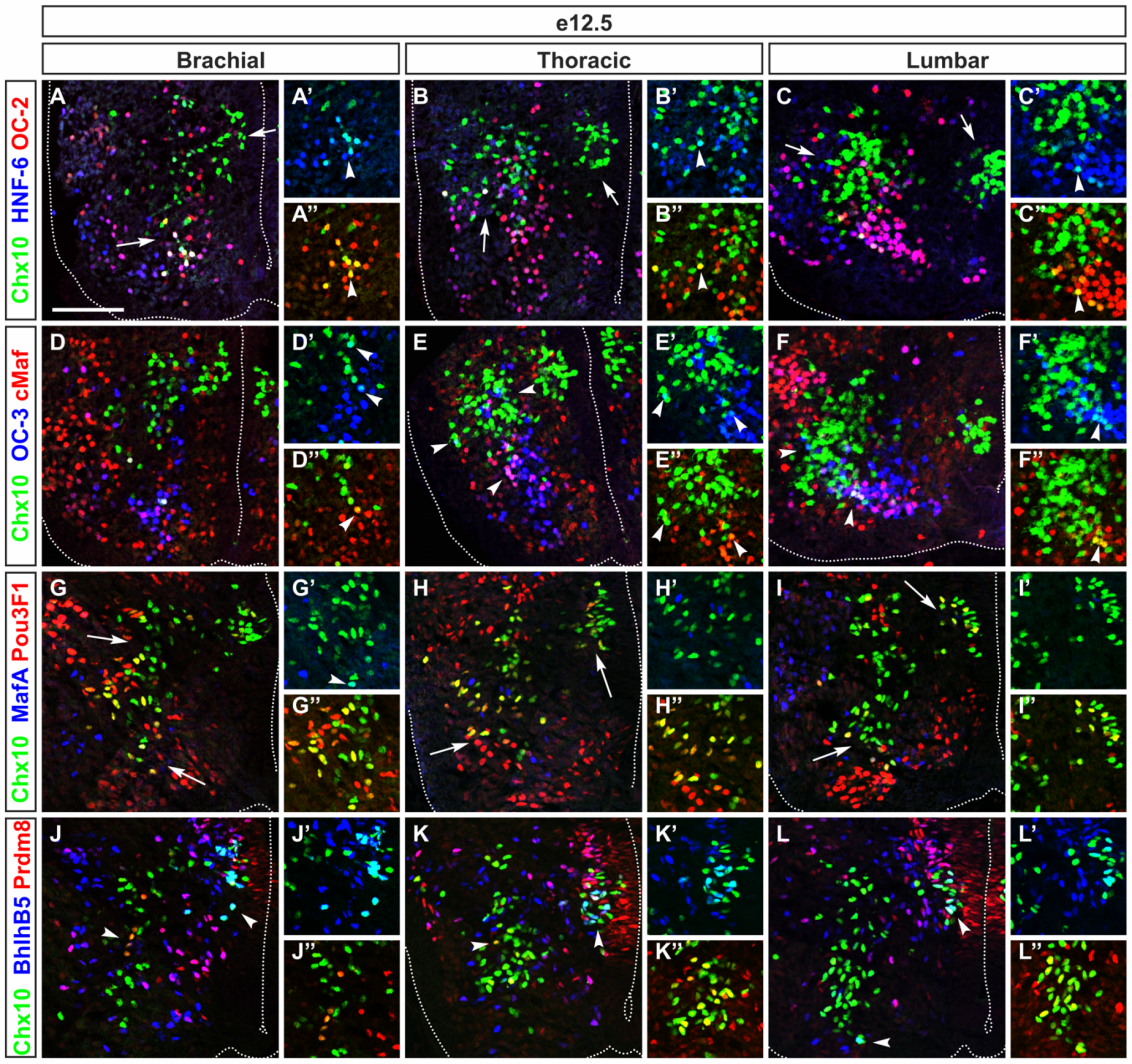
V2a interneurons subdivisions are differentially distributed along the rostrocaudal axis of the spinal cord. (A–A″) Immunofluorescence labeling on transverse section at brachial levels of e12.5 embryonic spinal cord shows that V2a interneurons subdivide into medial and lateral groups (arrows). Lateral group comprise small ventral subsets (arrowhead) that contain HNF-6 (cyan), OC-2 (yellow) or HNF-6 and OC-2 (white). (B–B″) At thoracic levels, OC-2^+^ subset (yellow) is absent, but HNF-6^+^/OC-2^+^ subset (white) is detected dorsally to motor neurons (arrowhead) while the HNF-6^+^ subset (cyan) is expanded in lateral group (left arrow). (C–C″) At lumbar levels, the amount of HNF-6^+^/OC-2^+^ (white) indicated by arrowhead or of HNF-6^+^ V2a neurons (cyan) decreases at lateral group (left arrow). (D–D″) At brachial levels, a ventral subset of V2a contains cMaf and OC-3 (white), while OC-3 is present in subsets of V2a (cyan) located laterally to newly-born V2a interneurons (arrowheads). (E–E″) At thoracic levels, V2a that contain OC-3 (in cyan) are less numerous while cMaf^+^/OC-3^+^ subset (white) located ventrally is still present (arrowheads). (F–F″) At lumbar levels, the amount of V2a containing OC-3 (cyan) is increased (left arrowhead) and this subset is located dorsally to the cMaf^+^/OC-3^+^ division (white) as indicated by bottom arrowheads. (G–G″) At brachial levels, Pou3F1 defines a large subset of V2a (yellow) located laterally and another one close to the newly-born V2a interneurons (arrows). MafA is detected in a few V2a cells that co-express Pou3F1 (white) as indicated by arrowhead. (H–H″) At thoracic levels, Pou3F1 is detected in majority of V2a interneurons (arrows) whereas MafA remains restricted to a few cells. (I–I″) At lumbar levels, Pou3F1 is present in a subset of V2a (yellow) located ventrally and close to motor neurons (arrow). MafA define a small subset of V2a that co-express Pou3F1 (white) and is located more ventrally, close to the floor plate (arrow). (J–J″) At brachial levels, BhlhB5 define a subset of newly-born V2a interneurons (cyan) indicated by right arrowhead while Prdm8 is detected in another subset (yellow) located in the dorso-ventral flux of V2a interneurons as shown by left arrowhead. Prdm8 and BhlhB5 (white) are detected in a few V2a close to the central canal. (K–K″) At thoracic levels, this population is expanded (arrowheads). (L–L″) At lumbar levels, BhlhB5 is detected in subsets of V2a interneurons including newly-born V2a (cyan) and V2a in the dorso-ventral flux (arrowheads). Prdm8 is also detected in subsets within the dorso-ventral flux and in newly-born V2a (yellow) and is present in some BhlhB5^+^ V2a (white). Scale bar = 100 µm.

**Figure 5:**
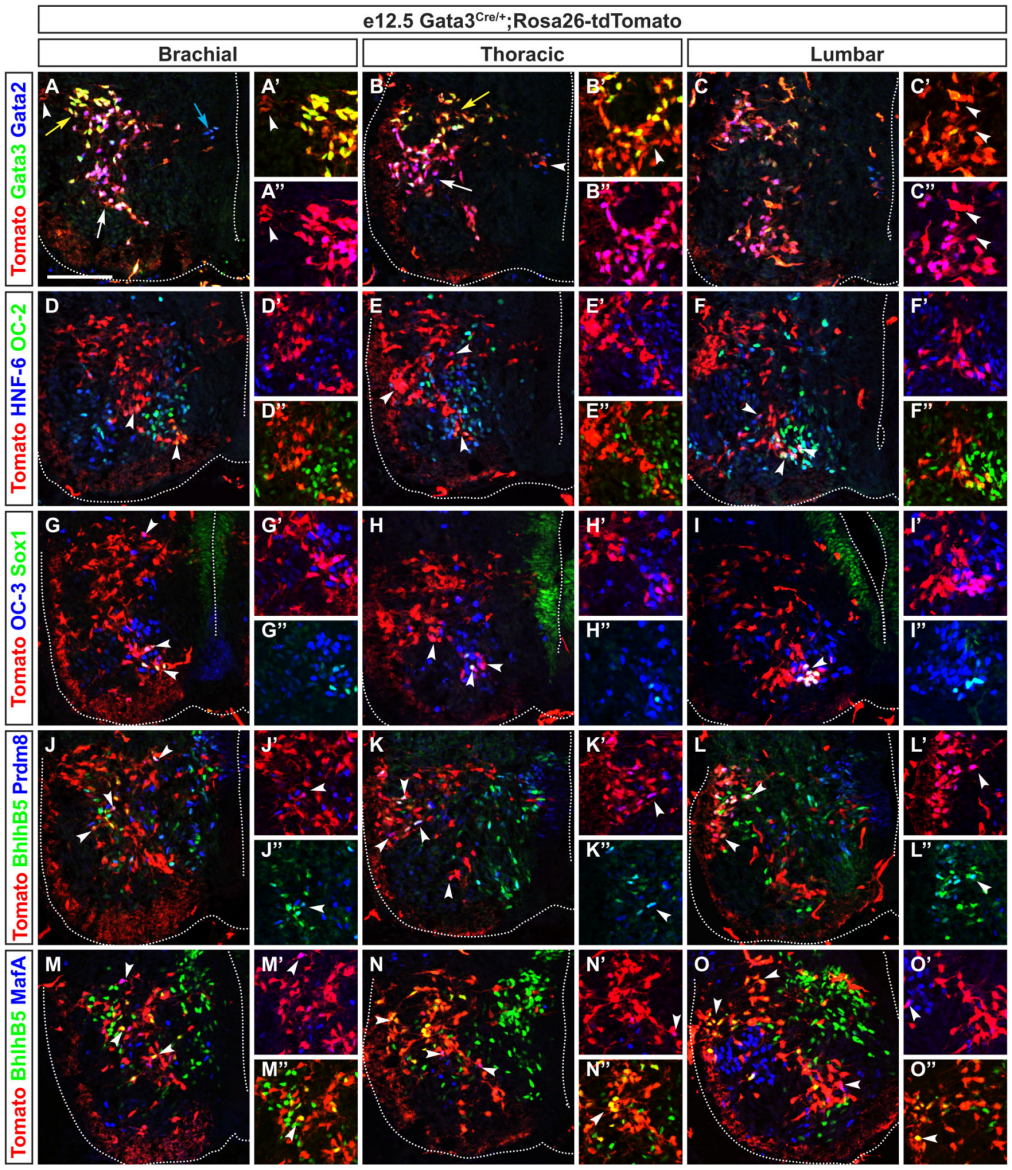
Differential distribution of V2b interneuron subdivisions along the rostrocaudal axis of the spinal cord. Immunofluorescence labeling on transverse section at brachial levels of the spinal cord of Gata3^Cre/+^Rosa26^floxstop-tdTomato^ embryo at e12.5. (A–A″) V2b interneurons subdivide into a dorsal subset that contains high levels of Gata3 (yellow) indicated by yellow arrow, a ventral subset that contains Gata2 (magenta) indicated by white arrow and a subset of newly-born V2b wherein high Gata2 and low Gata3 levels are detected (blue arrow). (B–B″) At thoracic levels, low content in Gata2 (blue) is detected in a dorsal Gata3^+^ subset (yellow) indicated by yellow arrow while low content in Gata3 is detected in the Gata2^+^ subset (white) located ventrally (white arrow). (C–C″) At lumbar levels, Gata2 and Gata3 are present in a dorsal and in a ventral subset of V2b interneurons (white). (D–D″) At brachial levels, HNF-6 or OC-2 are present (magenta or yellow) or co-detected (white) in subsets of V2b interneurons (arrowheads). (E–E″) Similarly, at thoracic levels, HNF-6 and OC-2 are co-detected in a ventral subset of V2b interneurons (white) as indicated by bottom arrowhead. OC-2 is present in a few V2b (yellow) while HNF-6 defines a small lateral subset (magenta) indicated by left and top arrowheads. (F–F″) At lumbar levels, HNF-6 and OC-2 are also co-detected in a ventral subset of V2b interneurons (white) indicated by bottom arrowheads while OC-2 (yellow) or HNF-6 (magenta) alone defines small subsets of V2b (left arrowhead). (G–G″) At brachial levels, OC-3 is present in all V2c interneurons (bottom arrowheads), which contain Sox1 (white), and in a subset of V2b (magenta) located dorsally to V2c neurons. (H–I″) Similarly, at thoracic and lumbar levels, all V2c are OC-3^+^ (white) and OC-3 is also present in another subset of V2b cells (arrowheads). (J–J″) At brachial levels, BhlhB5 defines a subset of V2b (yellow) close to motor neurons (left arrowhead). BhlhB5 and Prdm8 are co-detected in a few of these cells (white) as indicated by top arrowheads. (K–K″) At thoracic levels, BhlhB5 and Prdm8 are present in a subset of V2b (white) located dorsally to the motor neurons (left arrowheads). In addition, Prdm8 is detected in a small subset of V2b (magenta), which is located dorsally to BhlhB5^+^/Prdm8^+^ V2b subset (left and bottom arrowheads). (L–L″) At lumbar levels, BhlhB5 and Prdm8 are present in a subset of V2b (white) located dorsally to the motor neurons (top arrowhead). Similarly to thoracic levels, Prdm8 is observed in subset of V2b (magenta) laterally and close to BhlhB5^+^/Prdm8^+^ subset of V2b interneurons (bottom arrowhead). (M–M″) At brachial levels, MafA is detected in a small subset of V2b (magenta) indicated by top arrowhead whereas BhlhB5 defines another subset of V2b (yellow) indicated by arrowheads. (N–N″) At thoracic levels, the amount of V2b neurons that are MafA^+^ (magenta) is decreased (right arrowhead) compared to brachial levels. V2b subset expressing BhlhB5 (yellow) is detected dorsally to the motor neurons (left arrowheads). (O–O″) At lumbar levels, fewer MafA^+^ V2b cells (magenta) are observed than at brachial or thoracic levels (right arrowhead) while BhlhB5^+^ V2b subset (yellow) is located dorsally to the motor neurons and close to the basal lamina (left arrowheads). Scale bar = 100 µm.

At brachial levels of the spinal cord, V2a INs segregated into a medial and a ventral subdivision (arrows in Fig. 4A–A″). The three members of the Onecut family were present in a subset of the ventral subdivision in a partially overlapping pattern (arrowheads in Fig. 4A–A″, D–D″, Fig. 7K–L). Conversely, Pou3F1 was detected in a large subset of V2a located laterally and ventrally (arrows in Fig. 4G–G″, Fig. 7M). Prdm8, Bhlhb5, cMaf and MafA further defined subsets of the lateral V2a neurons (Fig. 4D–D″, 4G–G″, 4J–J″, Fig. 7L–N). To characterize V2b subsets, we used Gata3^Cre/+^;Rosa26^floxstop-tdTomato^ mouse embryos in which V2b and V2c are permanently labeled by the expression of tdTomato. V2b migrated laterally from their progenitor domain toward the MNs of the LMC, then turned toward a dorsal or a ventral direction to medially surround the MNs (Fig. 5A). Three V2b subsets were identified based on their distribution, on the levels of Gata2 and Gata3, and on their location along the dorso-ventral axis (Fig. 5A–A″). Gata2 and Gata3 were codetected in a large subpopulation of V2b, located medially to the MNs (white arrow in Fig. 5A–A″, Fig. 7O). Gata2 alone was detected in a small subpopulation of newly-born V2b close to the progenitor domain (blue arrow in Fig. 5A–A″) whereas a dorso-lateral subset of V2b was defined by a high levels of Gata3 (Gata3^high^) associated with low levels of Gata2 (Gata2^low^) (yellow arrow in Fig. 5A–A″ and Fig. 7O). Surprisingly, we also identified a small fraction of V2b that did not co-express neither Gata2 nor Gata3 (arrowheads in Fig. 5A–A″ and Fig. 7O). In the ventral part of the Gata2^+^/Gata3^+^ population, part of the V2b subsets contained Onecut transcription factors (arrowheads in Fig. 5G–G″, Fig. 7P–Q and data not shown). Although BhlhB5 was reported to not to be present in V2b at e10.5 [53], a subset of V2b located at the centre of the Gata2^+^/Gata3^+^ subpopulation and close to the MNs contained BhlhB5 (arrowheads in Fig. 5J–J″, Fig. 7R). Prdm8 was present with Bhlhb5 in a part of these cells and was also detected in a distinct V2b subset (arrowheads in Fig. 5J–J″, Fig. 7R). A last subset was characterized by the presence of MafA^+^ (Fig. 5M–M″, Fig. 7S). Finally, V2c INs characterized by the presence of Sox1 were located ventrally to the Gata2^+^/Gata3^+^ V2b population and contained HNF-6, OC-2 and OC-3 (arrowheads in Fig. 5G–G″, Fig. 7P–Q, and data not shown).

The distribution of V2a subsets was relatively stable at thoracic and at lumbar levels compared to brachial regions (Fig. 4B–C″, 4E–F″, Fig. 7K–N), with the exception of the Pou3F1 subset that expanded at thoracic levels and of the Prdm8^+^ V2a neurons that were more numerous at thoracic and lumbar levels (Fig. 4H–I″, 4K–L″, Fig. 7M–N). Similarly, V2b and V2c subsets were very homogenous along the rostrocaudal axis of the spinal cord (Fig. 5, Fig. 7O–S and data not shown).

Taken together, these observations showed that V2 subpopulations can be divided into multiple subsets characterized by the combinatorial presence of HNF-6, OC-2, OC-3, MafA, cMaf, Pou3F1, Prdm8 or Bhlhb5. These subsets exhibited a rather homogenous distribution along the rostrocaudal axis of the spinal cord.

### Differential distribution of V3 interneuron subsets along the rostrocaudal axis of the spinal cord

**Figure 6:**
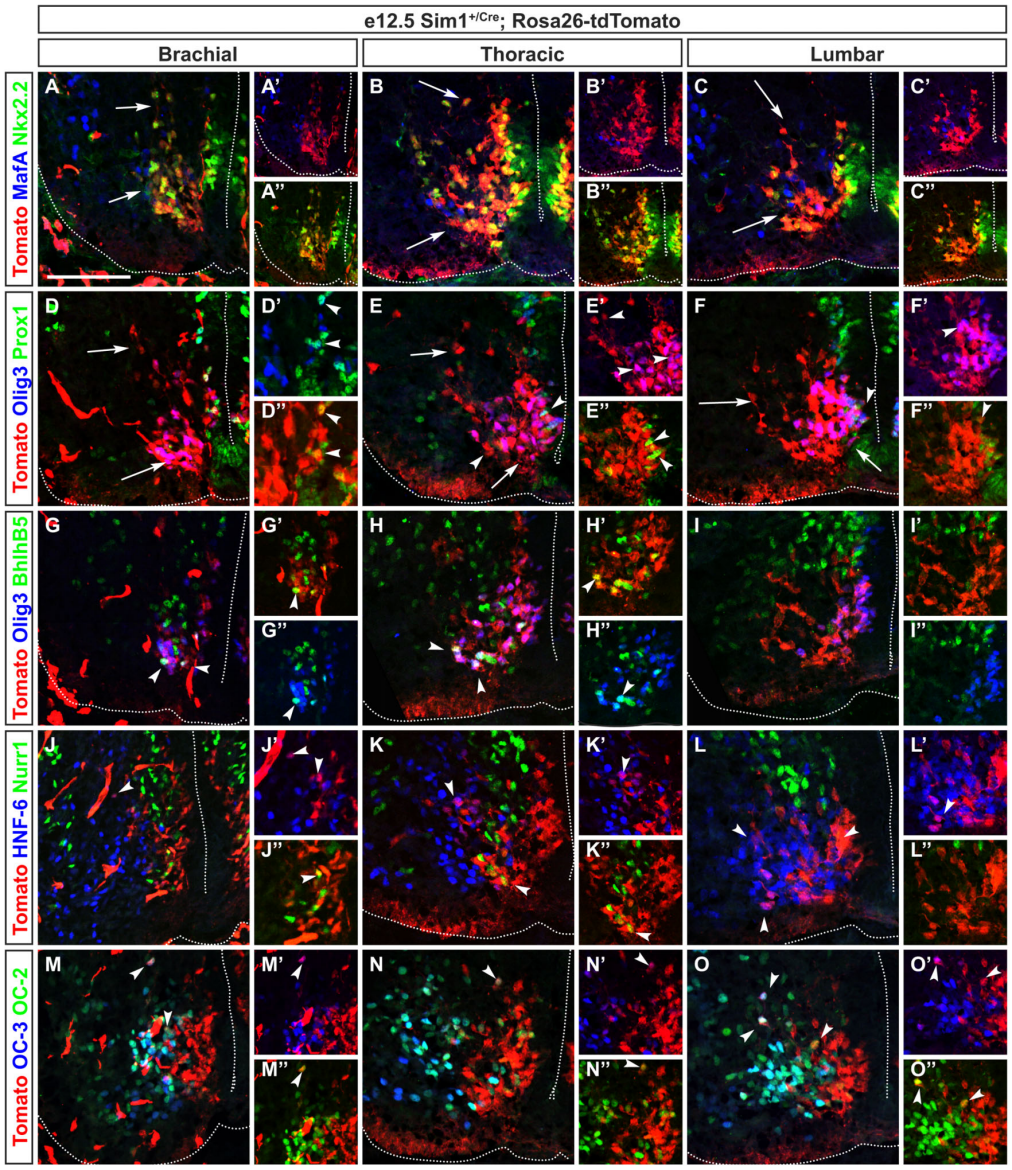
V3 interneuron subsets are differentially distributed along the rostrocaudal axis of the spinal cord. Immunofluorescence labeling on transverse section at brachial levels of the spinal cord of Sim1^Cre/+^;Rosa26^floxstop-tdTomato^ embryo at e12.5. (A–A″) All the V3 interneurons contain Nkx2.2 (yellow) and subdivide in 2 groups (arrows), while MafA (magenta) is present in a few V3 cells. (B–B″) At thoracic levels, a majority of V3 neurons are labeled for Nkx2.2 (yellow) and very few of them contain MafA (white). (C–C″) Similarly, at lumbar levels, almost all V3 neurons (in red) are labeled for Nkx2.2 (yellow) and MafA define a very small subset of V3 neurons (white or magenta), located close to the floor plate. (D–D″) At brachial levels, a majority of V3 neurons located ventrally, possibly corresponding to V3_V_, contain Olig3 (magenta) indicated by bottom arrow. In contrast, Prox1 is detected in very few V3 neurons (yellow or white) indicated by arrowheads. (E–E″) At thoracic levels, a large subset of V3 neurons located ventrally is defined by Olig3 (magenta) indicated by bottom arrow, a part of them containing Prox1 (white) indicated by right arrowhead. (F–F″) At lumbar levels, Olig3 is detected in more ventral V3 neurons (magenta) indicated by right arrow but not in V3 cells that migrate more dorsally indicated by left arrow, possibly corresponding to V3_D_. Prox1 is present in some V3_V_ neurons (yellow or white) as shown by arrowhead. (G–G″) At brachial levels, BhlhB5 is detected in some V3_V_ Olig3^+^ neurons (white) indicated by arrowheads. (H–H″) At thoracic levels, the amount of V3_V_ neurons that contain BhlhB5 (white) is increased (arrowheads). (I–I″) At lumbar levels, BhlhB5 is detected neither in V3_V_ nor V3_D_. (J–J″) At brachial levels, HNF-6 is detected in migrating V3_D_ neurons (magenta) indicated by arrowhead while Nurr1 is present in very few V3_V_ neurons (yellow). (K–K″) Similarly, at thoracic levels, HNF-6 is observed in migrating V3_D_ neurons (magenta) indicated by left arrowhead whereas Nurr1 is present in a small subset of V3_V_ neurons (yellow) indicated by right arrowhead. (L–L″) At lumbar levels, HNF-6 is detected in migrating V3_D_ neurons (magenta) indicated by left arrowheads while Nurr1 is absent from V3 neurons (see right arrow). (M–M″) At brachial levels, OC-2 and OC-3 are co-detected in all V3_D_ neurons (white) indicated by top arrowhead while a few V3_V_ are labeled for OC-2 only (yellow) as indicated by bottom arrowhead. (N–N″) Similarly, at thoracic levels, OC-2 and OC-3 are present in all V3_D_ neurons (white) indicated by arrowhead while OC-2 alone is detected in a small subset of V3_V_ (yellow). (O–O″) At lumbar levels, a large subset of V3_D_ contain OC-2 and OC-3 (white) indicated by top arrowheads, but OC-2 is also present in subsets of V3_D_ and V3_V_ neurons (yellow) as shown by right arrowhead. Scale bar = 100 µm.

V3 INs characterized by Sim1 and Nkx2.2 expression arise from p3 progenitors defined by Nkx2.2 [86], [87]. These excitatory commissural neurons diversify into V3_D_ and V3_V_ according to their location and their respective electrophysiological characteristics [52]. However, markers for V3_D_ or V3_V_ cells have not been identified to date. To analyze the V3 INs, we relied on a Sim1^Cre/+^;Rosa26^floxstop-tdTomato^ mouse line in which all V3 derivatives are permanently labeled by the expression of tdTomato. V3 INs initially gathered close to the floor plate and the central canal, and migrated according to a ventral to dorso-lateral stream and along a ventral to dorsal stream close to the midline (arrows in Fig. 6A–C).

**Figure 7:**
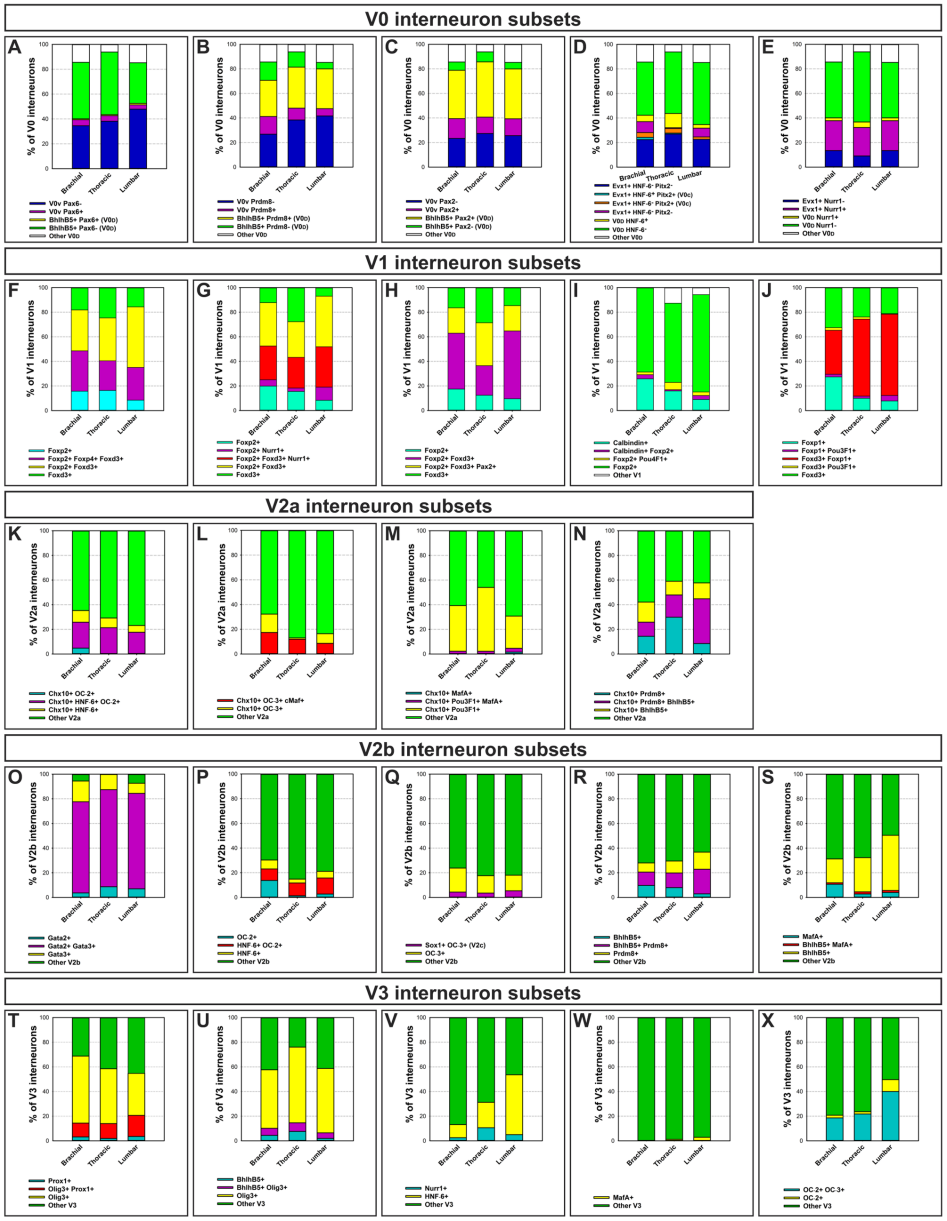
Quantification of ventral interneuron subsets along the anterposterior axis of the spinal cord. (A–X) Histograms of quantification of V0, V1, V2a, V2b and V3 interneuron subsets at brachial, thoracic and lumbar levels of the spinal cord of mouse embryos at e12.5 show distinct distribution and proportion of different subsets within each interneuron cardinal classes. Histograms are normalized according to the total amount of cells in each ventral IN population, which corresponds to 100% (n = 3).

First, we confirmed that Nkx2.2 was maintained in all V3 derivatives along the rostrocaudal axis of the spinal cord at e12.5 (Fig. 6A–C) [88]. Then, at brachial levels, we observed that Olig3, a transcription factor which is present in p0, p2 and p3 progenitor domain [74], was also detected in a large subset of V3 INs located ventrally and close to the central canal (arrow in Fig. 6D–D″ and Fig. 7T), but not in more dorsal and/or migrating V3 INs (arrow in Fig. 6D′, 6E′, 6F′). These cells could correspond to V3_V_ INs. This population further subdivided into subsets characterized by the presence of Prox1, BhlhB5 and Nurr1 (arrowheads in Fig. 6D–D″, 6G–G″, 6J–J″, Fig. 7V). By contrast, members of the Onecut transcription factor family were detected in subsets of V3 INs located in a dorso-lateral stream, likely corresponding to V3_D_ cells (arrowheads in Fig. 6J–J″, 6M–M″, Fig. 7V–W) and also in a few V3 neurons close to the floor plate that correspond to V3_V_ cells.

At thoracic and lumbar levels, the location of V3 INs did not change significantly compared to brachial levels. Nonetheless, the amount of V3_D_ INs gradually increased at the expense of the V3_V_ INs that contained Olig3 (Fig. 6D–I″, Fig. 7T–U). In addition, subsets containing Nurr1 or Bhlhb5 were expanded at thoracic levels (arrowheads in Fig. 6K–K″, Fig. 6H–H″, Fig. 7U–V).

Together, these data indicate that the topographic distribution of V3 INs did not significantly differ at limbs or thoracic levels but that V3 subsets are differentially distributed along the rostrocaudal axis of the developing spinal cord. Most of these subsets are characterized by distinct combinations of transcription factors.

## Discussion

Despite the identification of four cardinal classes of ventral spinal INs and the discovery of some of the molecular and genetic mechanisms that control their specification, little is known about their respective spatial distribution along the rostrocaudal axis of the spinal cord and their diversification into multiple subsets. Here, we provided evidence that each class of ventral INs displays distinct columnar organization that varies at brachial, thoracic and lumbar levels of the developing spinal cord. In addition, we showed that the V0, V1, V2 and V3 cardinal classes of INs can be subdivided into several subpopulations or subsets, based on the combined expression of multiple transcription factors (Table 3). Hence, these findings provide a basis for a better characterization of the diversification of embryonic ventral INs, and for investigating the physiology and function of ventral INs subsets in the spinal motor circuits.

**Table 3:** Summary of ventral interneuron subsets at e12.5.

Interneuron Classes	Subpopulations		Subset markers
**V0**	V0_D_	BhlhB5	Pax2>Prdm8>Nurr1
	V0_V_	Evx1	Pax2>OC3>Nurr1>Prdm8>Pax6
			HNF-6/OC-2>MafA
	V0_CG_	Evx1, Pitx2	HNF-6
**V1**	Renshaw cells	Calbindin, OC^a^,	
		Foxd3, En1, MafB	
	Ia INs	Foxp2, Foxd3, En1	Foxp4>Pax2>Arx>Nurr1
			BhlhB5,Prdm8>Prdm8>Pou4F1
	Other V1	Foxd3, En1	Foxp1>Pou3F1
			Nurr1>BhlhB5>Prdm8
**V2**	V2a	Chx10	BhlhB5>Pou3F1
			OC^a^>Prdm8>MafA>cMaf
	V2b	Gata3, Gata2	BhlhB5>BhlhB5/Prdm8>Prdm8
			OC^a^>MafA/MafB
	V2c	Sox1, OC^a^	
**V3**	V3_D_	OC^a^	
	V3_V_	Olig3	Prox1>BhlhB5
			Nurr1

Summary of the subdivisions of ventral interneuron classes, according to the expression of the studied markers (from more numerous to less numerous cells).

a The three Onecut factors.

Supporting Information Legends.

### Topographic and columnar organization of ventral spinal INs

The differential distribution of ventral IN populations and subsets along the rostrocaudal axis of the spinal cord likely correlates with the various functions of local spinal neuronal circuits that regulate body movements or locomotive behaviors. Indeed, thoracic circuits ensure control of body wall musculature, whereas brachial and lumbar neuronal populations additionally provide regulation of limb movements. Consistently, the composition in ventral IN subsets varies along the rostrocaudal axis of the spinal cord, suggesting that the local pre-motor circuits at brachial, thoracic or lumbar levels are shaped to support these distinct activities. The exact position and contribution of each IN subset within these pre-motor circuits, including its direct or indirect relationship to MNs, remains to be determined. Surprisingly, the differences in the composition of vIN subsets between brachial and lumbar levels were as important as between these limb levels and thoracic levels. This suggests that the organization of the locomotor circuits and the strategies to control limb movements are quite different between the brachial and the lumbar region. This observation may be correlated with the distinct composition in motor pools between these two regions [25], [89], [90].

Previous studies have established that spinal MNs also exhibit a differential organization along the rostrocaudal axis of the spinal cord [22], [91]. Studies of the developmental mechanisms that control the topographic and columnar organization of MNs reveal roles for extrinsic factors such as RA, FGFs and Wnts in regulating Hox gene expression [15], [16], [21], [22], [23]. As per motor neurons, the topographic organization of ventral INs may be regulated by RA, FGFs or Wnts, either directly or through combined activities of Hox genes. Indeed, RA from the paraxial mesoderm appears to be sufficient to specify and promote the generation of V0, V1 and V2 INs [78], [92], [93], [94]. In addition, LMC MNs constitute another source of RA that could influence the differentiation and distribution of V0, V1 and V2 INs at brachial and lumbar levels [23], [95], [96], [97]. However, the potential role of RA in the distribution of V0, V1 and V2 INs along the rostrocaudal axis has not been investigated yet. Previous studies have also shown that the Hox genes involved in the rostrocaudal patterning of MNs and in the specification of motor neuron pools, namely Hoxc6 and Hoxc8 at brachial levels, Hoxc9 and Hoxd9 at thoracic levels and Hoxa10, Hoxc10 and Hoxd10 at lumbar levels, are also expressed in ventral INs [27], [96], [98]. Moreover, the Hox co-factor Foxp1 is expressed in V1 INs, while Pbx1 and Prep/Meis are more broadly expressed in ventral INs [99], [100], [101], [102]. This raises the possibility that these co-factors partner with Hox proteins to differentially regulate ventral IN diversification. The Hox genes may therefore coordinately regulate rostrocaudal distribution and columnar organization of ventral INs and of MNs in the developing spinal cord.

### Control of the diversification of ventral INs

With the exception of studies that show Scl and Dll4/Notch signaling control the generation of V2a and V2b cells from V2 progenitors [47], [85], [103], we still know very little about the molecular mechanisms and factors that regulate ventral INs diversification. Several studies have unveiled that extrinsic factors including Sonic hedgehog, BMPs, FGFs, Wnts or RA are involved in the specification and differentiation programs of several neuronal populations within the spinal cord [104], [105], [106]. Our study describes a number of developmental regulated transcription factors that subdivide three cardinal classes of ventral INs into several subsets. Interestingly, some of these factors might be downstream of FGF or RA signaling [25], [55], [90], [98]. Indeed, the expression of Nurr1, which labels some subsets in V0_V_, V1 non-RC neurons or V3_V_ INs, is regulated by FGFs in the mesencephalon [107], [108], [109] and by RA signaling in the ventral spinal cord [53]. Similarly, BhlhB5, which defines several subsets within V0, V1, V2b and V3 INs, is modulated by RA in the same tissue [53]. Furthermore, Pou3F1, which defines subsets within V1 and V2a INs, is regulated by Hoxc8 in the motor pool that innervates the flexor carpi ulnaris muscle [25]. The Onecut factors, which are detected within Nkx6.1^+^, Pea3^+^ or Pou3F1^+^ motor pools [55] and may therefore be downstream of Hox genes, are also expressed within several subsets of V0, V2 and V3 INs (at brachial or lumbar levels). Thus, the mechanisms that ensure proper rostrocaudal patterning of the motor neurons, including FGF or RA pathways and Hox genes, may additionally contribute to the diversification of ventral spinal IN subsets, as previously suggested for Hox4-8 genes [27].

In addition, the transcription factors identified in the present study as markers of ventral IN subsets may participate in the control of their diversification. Onecut factors have been recently shown to participate in the development of RCs [44]. Similarly, BhlhB5 plays a crucial role in the differentiation of early-born V2 INs [53]. Surprisingly, we identified BhlhB5 as a marker of most of the V0_D_ INs at e12.5, although previous studies have reported that BhlhB5 is not expressed in V0 cells at e10.5 [53], [68], [69]. This suggests that Bhlhb5 expression may be activated in a majority of V0_D_ INs later than e10.5. Alternatively, as recently demonstrated for V1 INs development that involves successive waves of neurogenesis [43], [44], V0 progenitors may give rise to V0_V_ (devoid of Bhlhb5) prior to V0_D_ cells (that express BhlhB5). Given the importance of Bhlhb5 for the development of other spinal populations [53], [70], [110], it also indicates that Bhlhb5 could participate in further diversification of V0_D_ cells. In addition, we identified a large BhlhB5^+^ subset within the V2b subpopulation whereas it has been reported that BhlhB5 is selectively expressed at e10.5 in V2a but not in V2b neurons [53]. Further analyses of BhlhB5 function using loss or gain-of-function experiments are needed to clarify the role of BhlhB5 during V0_D_ and V2 INs development. Finally, Nurr1 is expressed in glutamatergic cortical neurons [76], [77] and in glutamatergic spinal INs (unpublished data), which suggests that Nurr1 may participate in the differentiation of glutamatergic neurons including V0_V_ and V3 INs [71].

Furthermore, most of the identified markers of IN subsets are expressed in a combinatorial manner and may interact to promote specific neuronal fate. For example, BhlhB5 and Prdm8, which shares numerous target genes, cooperate during differentiation of dI6, V1 and V2a INs [44], [53], [69] and of dorsal INs [70], [110]. We have shown that Prdm8 is co-expressed with BhlhB5 in some subsets of V0_D_, V1 and V2 INs. However, we also identify Prdm8^+^ subsets that do not express BhlhB5 and vice versa. Among the identified common target genes of BhlhB5 and Prdm8, the type II cadherin-11 (Cdh-11) [70] has been reported to regulate different aspects of spinal motor neuron development including differentiation, columnar organization [111], axonal growth, migration and fasciculation [112], [113]. At e12.5, Cdh-11 is transiently expressed in ventral INs [111]. Hence, upon BhlhB5 and Prdm8 regulation, Cdh-11 may participate in the diversification of ventral INs subsets.

Finally, numerous studies have revealed that Foxp factors control different steps of neuronal development. For example, Foxp2 regulates neuronal network formation [114], [115], radial and/or tangential migration of cortical interneurons [116], neurite outgrowth [115] as well as differentiation of several neuronal populations within the ventral spinal cord including MNs, V0_V_ and V2 INs [114]. Foxp2 is also expressed from e11.5 within large subpopulations of non-RC V1 neurons including Ia INs [66] and may therefore control different aspects of their development. Likewise, Foxp4, which is present in Foxp2^+^ V1 cells, may participate in the regulation of differentiation, migration and axonal growth by cooperating with Foxp2 as recently described [117], [118], [119]. Foxp1 is also detected in subsets of V1 INs [66], [120], suggesting that different combinations of Foxp factors may regulate distinct aspects of V1 development including differentiation and migration as recently suggested [58], [66], [121].

### Conclusion and Perspectives

In this study, we showed that ventral spinal IN populations are differentially distributed along the rostrocaudal axis of the spinal cord, and can be subdivided into multiple subsets according to a combinatorial code of transcriptional regulators. This characterization constitutes a resource that will facilitate further studies aimed at understanding the mechanisms that drive ventral spinal IN diversity during embryonic development. It will also provide a foundation for establishing the relationship between these embryonic subsets and the multiple IN populations that populate the adult spinal cord.

Recent advances in the engineering of powerful genetically-modified biological tools, such as genetic lineage tracing mouse lines, modified trans-synaptic tracing viruses, conditional neuronal silencing and optogenetic systems [51], [122], [123] have greatly contributed to the improvement of our knowledge of neural circuitry and their physiological functions. Hence, our data will contribute to design strategies to genetically manipulate these neuronal subsets. Such experiments should allow to further decipher the organization of the spinal neural circuits that regulate locomotive behaviors and to elucidate in more details the different functions of these cells in the neonate and adult spinal cord.

## Supporting Information

Figure S1 **Topographic distribution of V0 interneurons at e12.5.** (A–C) Whole-mount immunofluorescence analysis on spinal cord of Dbx1^LacZ/+^ embryo at e12.5 shows differential distribution of V0 interneurons including V0_V_ (Evx1^+^/β-galactosidase^+^) in red or yellow and V0_D_ in green at brachial (A), thoracic (B) or lumbar (C) levels. The white arrow indicates ventral (V) to dorsal (D). Scale bar = 500 µm.
(TIF)

Figure S2 **Topographic distribution of V1 interneurons at e12.5.** (A–C) Whole-mount immunofluorescence analysis on spinal cord of embryo at e12.5 that shows differential distribution pattern of V1 interneurons including Renshaw cells (red) and V1 Foxp2^+^ interneurons (green) at brachial (A), thoracic (B) or lumbar (C) levels. White arrowhead indicates ventral (V) to dorsal (D). Scale bar = 500 µm.
(TIF)

Figure S3 **Topographic distribution of V2 interneurons at e12.5.** (A–C Whole-mount immunofluorescence analysis on spinal cord of embryo at e12.5 that shows differential distribution pattern of V2 interneurons including V2a (blue), V2b (red) and V2c (green) at brachial (A), thoracic (B) or lumbar (C) levels. White arrowhead indicates ventral (V) to dorsal (D). Scale bar = 500 µm.
(TIF)

Figure S4 **Topographic distribution of V3 interneurons at e12.5.** (A–C) Whole-mount immunofluorescence analysis on spinal cord of embryo at e12.5 that shows homogenous distribution pattern of V3 interneurons (white) at brachial (A), thoracic (B) or lumbar (C) levels. White arrowhead indicates ventral (V) to dorsal (D). Scale bar = 500 µm.
(TIF)

Figure S5 **V0_D_ interneurons contain BhlhB5 at e12.5.** (A–C″) Immunofluorescence labeling on transverse section at brachial (A–A″), thoracic (B–B″) and lumbar (C–C″) levels of the spinal cord of Dbx1^LacZ/+^ embryo at e12.5 shows that V0_D_ interneurons contain BhlhB5 (yellow) but not Lbx1^+^ (blue), a marker of dorsal interneurons. (D–F″) V0 interneurons that contain BhlhB5 (yellow) are not labeled for Foxd3^+^ (blue), a marker of V1 interneurons. Hence, in Dbx1^LacZ/+^ embryo, β-galactosidase is detected only in V0 interneurons and absent from V1 or dI6 neurons. Scale bar = 100 µm.
(TIF)

Figure S6 **Quantification of V0 or V1 subsets along the rostrocaudal axis of the spinal cord.** (A–D) Histograms of quantification of V0 and V1 interneuron subsets at brachial, thoracic and lumbar levels of the spinal cord at e12.5 show distinct distribution and proportion of different subsets within V0 and V1 interneuron cardinal class. Panels of 91 to 219 cells were counted for the V0 population, and 169 to 257 cells were counted for the V1 population, according to brachial, thoracic or lumbar levels. Histograms are normalized according to the total amount of cells in each ventral IN population, which corresponds to 100%. (n = 3).
(TIF)

Movie Collection S1 Vertical rotation of 3D reconstruction of flat mount “open-book” preparation at brachial (Movie S1), thoracic (Movie S2) or lumbar (Movie S3) levels of isolated spinal cord of Dbx1^LacZ/+^ embryo at e12.5 labeled for β-galactosidase (green) and Evx1 (red), corresponding to Fig. S1A. The movies focus on a single half of the spinal cord. Anterior is to the left, posterior is to the right. At the onset of the movies, ventral is to the top. (Movie S1) At brachial levels, V0_V_ interneurons (red or yellow) display a distribution pattern distinct from V0_D_ interneurons (green). V0 interneuron class exhibits a columnar organization. (Movie S2) At thoracic levels, V0_V_ interneurons (yellow) and V0_D_ interneurons (green) are differentially distributed compared to brachial section of the spinal cord. (Movie S3) At lumbar levels, a majority of V0_D_ interneurons (green) are distributed more ventrally than V0_V_ interneurons (yellow).
(ZIP)

Movie Collection S2 Vertical rotation of 3D reconstruction of flat mount “open-book” preparation at brachial (Movie S1), thoracic (Movie S2) or lumbar (Movie S3) levels of isolated spinal cord embryo at e12.5 labeled for V1 interneurons, corresponding to Fig. S2A. The movies focus on a single half of the spinal cord. Anterior is to the left, posterior is to the right. At the onset of the movies, ventral is to the top. (Movie S1) At brachial levels, Renshaw cells (red or magenta) form a large subset organized into a ventral column distinct from Foxp2^+^ V1 interneuron columns (green). (Movie S2) At thoracic levels, Renshaw cells (red or magenta) display a columnar organization but the size of this column is reduced compared to brachial levels. Similarly, size of Foxp2^+^ V1 interneurons (green) is decreased. (Movie S3) At lumbar levels, Renshaw cells (red or magenta) are more scattered compared to brachial or thoracic levels. By contrast, Foxp2^+^ V1 interneurons (green) are gathered in a compact and homogenous column.
(ZIP)

Movie Collection S3 Vertical rotation of 3D reconstruction of flat mount “open-book” preparation at brachial (Movie S1), thoracic (Movie S2) or lumbar (Movie S3) levels of isolated spinal cord embryo at e12.5 labeled for V2 interneurons, corresponding to Fig. S3A. The movies focus on a single half of the spinal cord. Anterior is to the left, posterior is to the right. At the onset of the movies, ventral is to the top. (Movie S1) At brachial levels, V2a interneurons (blue) are grouped into a distinct column that also contains some V2b interneurons (red). V2b interneurons are distributed into a large column located dorsally to V2a (interneurons) and a narrow column located ventrally to V2c interneurons (green). V2c interneurons are scattered in a column located ventrally to the V2a column. (Movie S2) At thoracic levels, V2a interneurons (blue) form a compact column that contains more V2b interneurons (red) compared to brachial region. V2b interneurons are organized into a large column located dorsally to V2a. V2c interneurons (green) are located ventrally to V2a interneurons within a narrow column that contains fewer V2b interneurons than at brachial levels. (Movie S3) At lumbar levels, V2b interneurons (red) are grouped into a column dorsal to the V2a (interneuron) column (blue) but also intermingle with V2a cells. V2c interneurons (green) gather in a narrow column located directly ventral to the V2a column.
(ZIP)

Movie Collection S4 Vertical rotation of 3D reconstruction of flat mount “open-book” preparation at brachial (Movie S1), thoracic (Movie S2) or lumbar (Movie S3) levels of isolated spinal cord embryo at e12.5 labeled for V3 interneurons, corresponding to Fig. S4A. The movies focus on a single half of the spinal cord. Anterior is to the left, posterior is to the right. At the onset of the movies, ventral is to the top. (Movie S1) At brachial levels, V3 interneurons (red) are distributed into a unique column. (Movie S2) At thoracic levels, V3 interneurons (red) are grouped into a compact column. (Movie S3) At lumbar levels, V3 interneurons (red) form a compact column, the thickness of which being reduced compared to brachial or thoracic levels.
(ZIP)
