# Supplementary figures and images for: Identification of Multiple Subsets of Ventral Interneurons and Differential Distribution along the Rostrocaudal Axis of the Developing Spinal Cord
Source: PLoS One. 2013 Aug 15;8(8):e70325. doi: 10.1371/journal.pone.0070325 (PMC3744532; doi:10.1371/journal.pone.0070325)

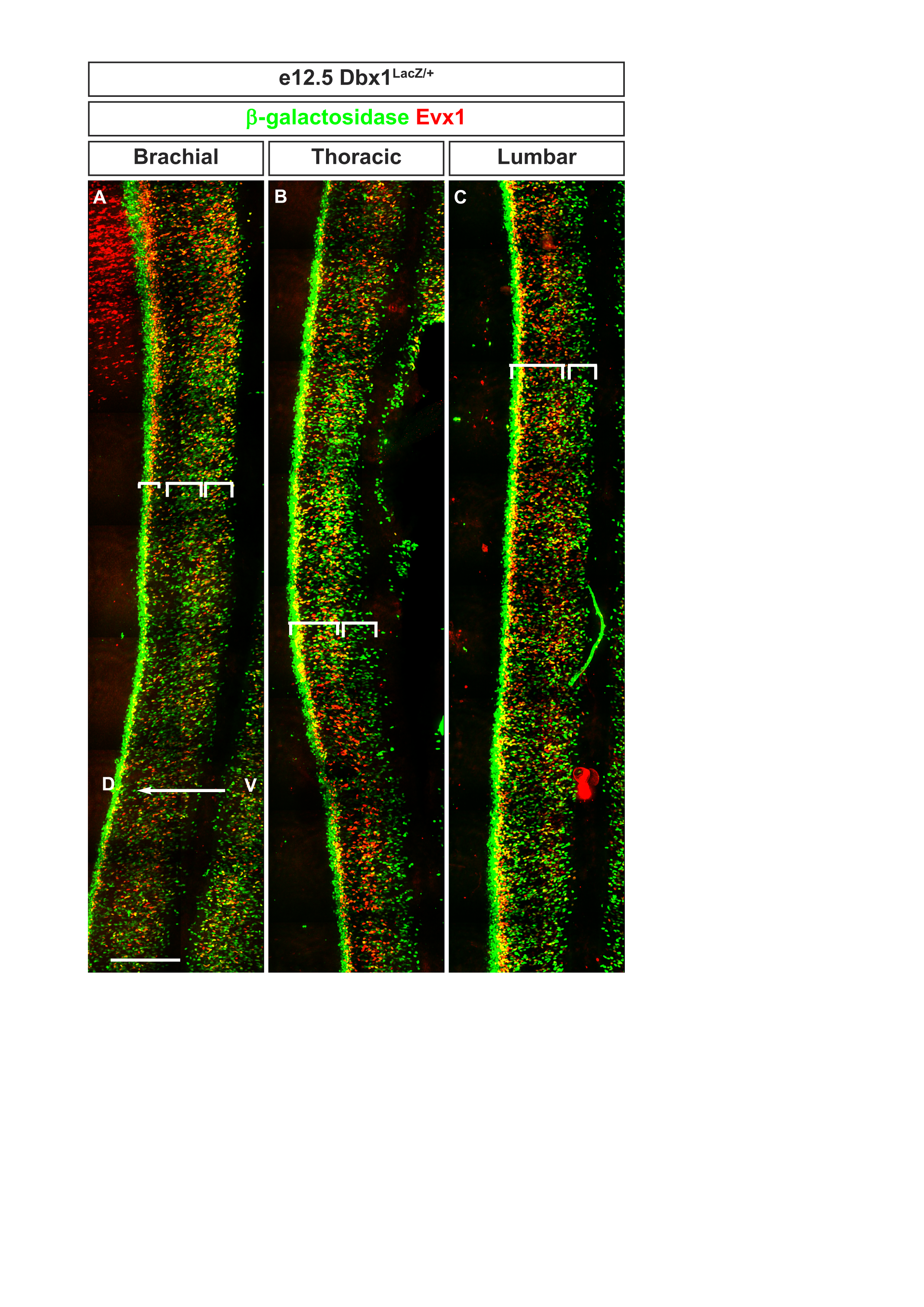

Supplement: Figure S1 — Topographic distribution of V0 interneurons at e12.5. (A–C) Whole-mount immunofluorescence analysis on spinal cord of Dbx1LacZ/+ embryo at e12.5 shows differential distribution of V0 interneurons including V0V (Evx1+/β-galactosidase+) in red or yellow and V0D in green at brachial (A), thoracic (B) or lumbar (C) levels. The white arrow indicates ventral (V) to dorsal (D). Scale bar = 500 µm. (TIF) [file pone.0070325.s001.tif]

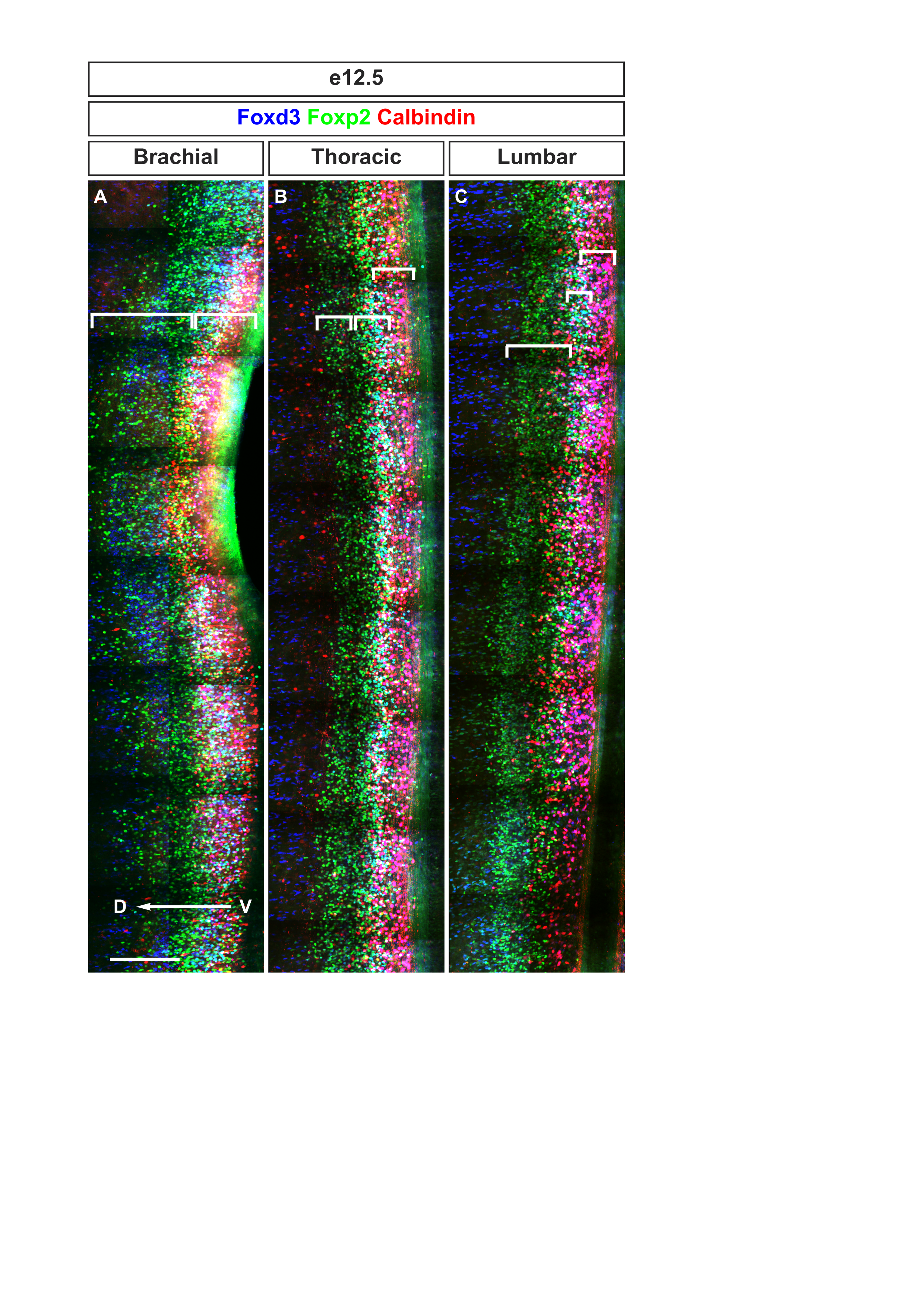

Supplement: Figure S2 — Topographic distribution of V1 interneurons at e12.5. (A–C) Whole-mount immunofluorescence analysis on spinal cord of embryo at e12.5 that shows differential distribution pattern of V1 interneurons including Renshaw cells (red) and V1 Foxp2+ interneurons (green) at brachial (A), thoracic (B) or lumbar (C) levels. White arrowhead indicates ventral (V) to dorsal (D). Scale bar = 500 µm. (TIF) [file pone.0070325.s002.tif]

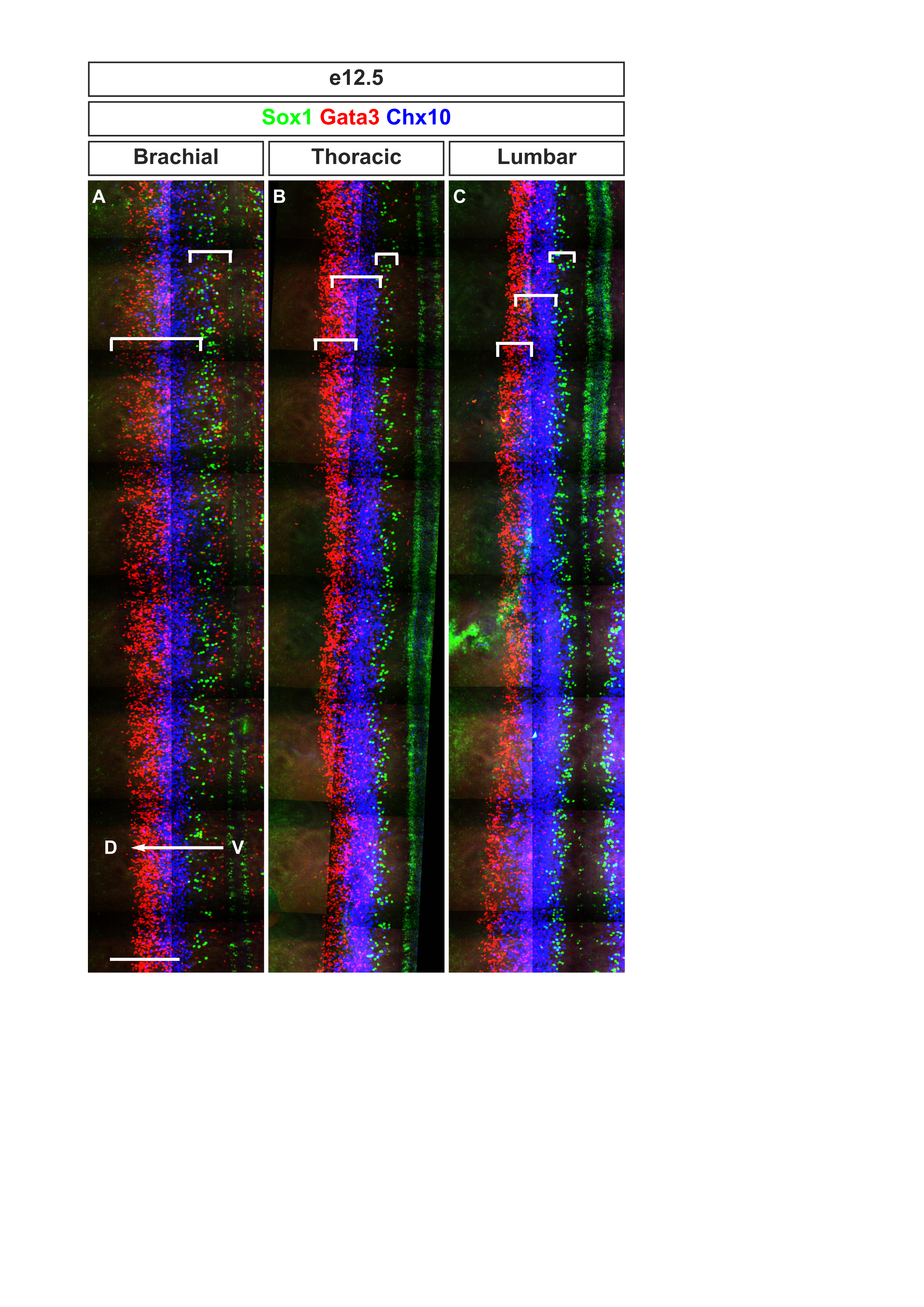

Supplement: Figure S3 — Topographic distribution of V2 interneurons at e12.5. (A–C Whole-mount immunofluorescence analysis on spinal cord of embryo at e12.5 that shows differential distribution pattern of V2 interneurons including V2a (blue), V2b (red) and V2c (green) at brachial (A), thoracic (B) or lumbar (C) levels. White arrowhead indicates ventral (V) to dorsal (D). Scale bar = 500 µm. (TIF) [file pone.0070325.s003.tif]

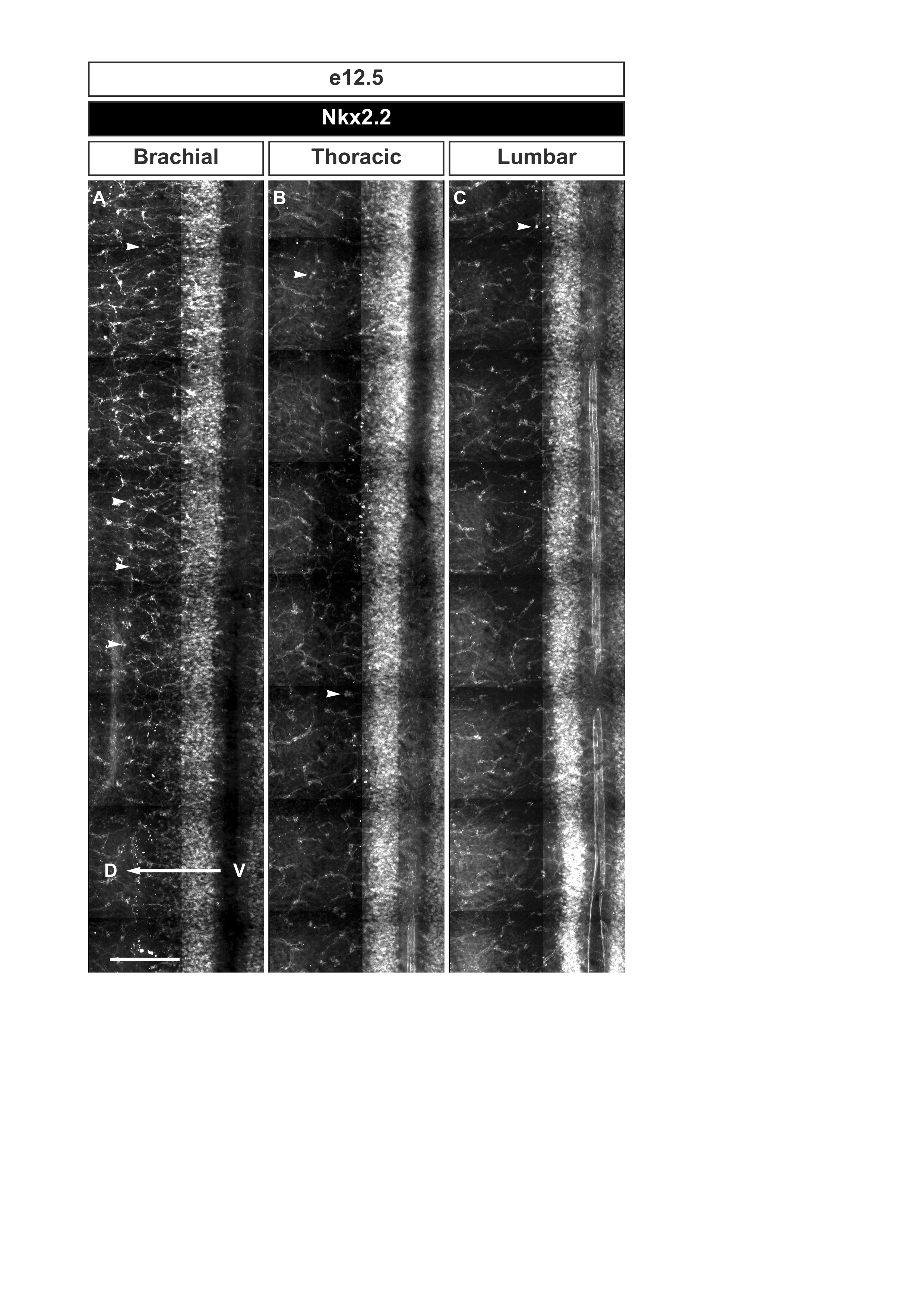

Supplement: Figure S4 — Topographic distribution of V3 interneurons at e12.5. (A–C) Whole-mount immunofluorescence analysis on spinal cord of embryo at e12.5 that shows homogenous distribution pattern of V3 interneurons (white) at brachial (A), thoracic (B) or lumbar (C) levels. White arrowhead indicates ventral (V) to dorsal (D). Scale bar = 500 µm. (TIF) [file pone.0070325.s004.tif]

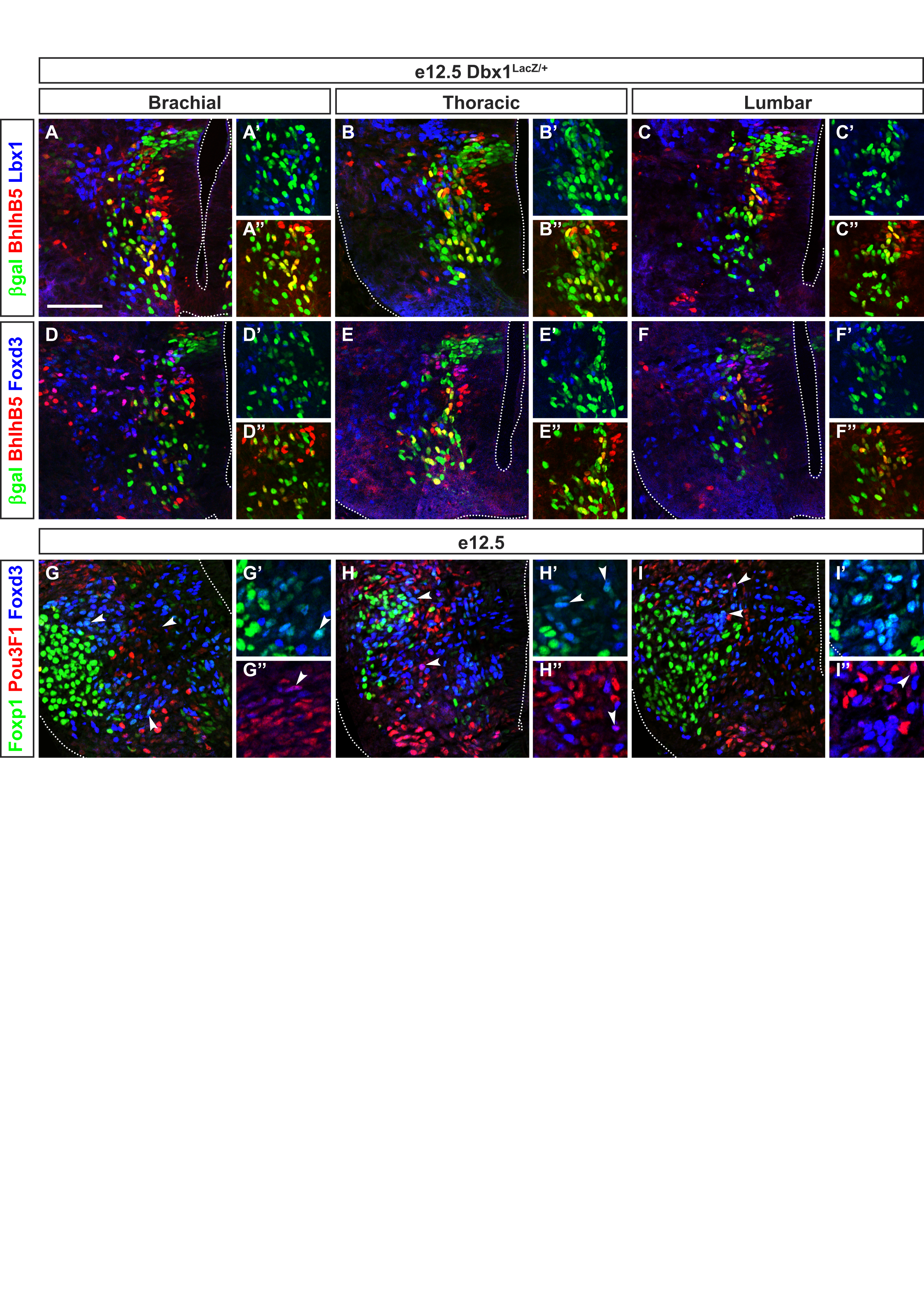

Supplement: Figure S5 — V0D interneurons contain BhlhB5 at e12.5. (A–C″) Immunofluorescence labeling on transverse section at brachial (A–A″), thoracic (B–B″) and lumbar (C–C″) levels of the spinal cord of Dbx1LacZ/+ embryo at e12.5 shows that V0D interneurons contain BhlhB5 (yellow) but not Lbx1+ (blue), a marker of dorsal interneurons. (D–F″) V0 interneurons that contain BhlhB5 (yellow) are not labeled for Foxd3+ (blue), a marker of V1 interneurons. Hence, in Dbx1LacZ/+ embryo, β-galactosidase is detected only in V0 interneurons and absent from V1 or dI6 neurons. Scale bar = 100 µm. (TIF) [file pone.0070325.s005.tif]

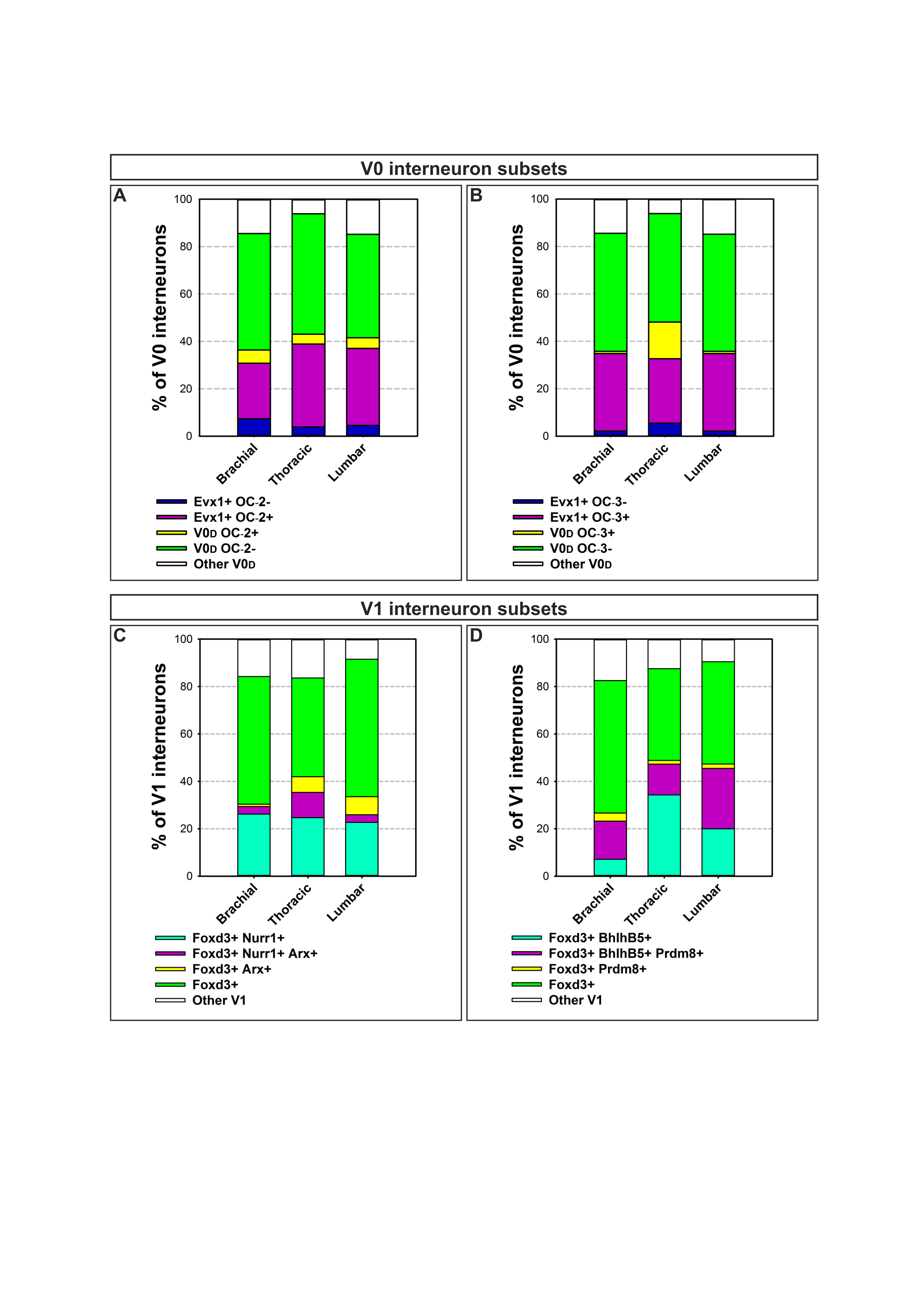

Supplement: Figure S6 — Quantification of V0 or V1 subsets along the rostrocaudal axis of the spinal cord. (A–D) Histograms of quantification of V0 and V1 interneuron subsets at brachial, thoracic and lumbar levels of the spinal cord at e12.5 show distinct distribution and proportion of different subsets within V0 and V1 interneuron cardinal class. Panels of 91 to 219 cells were counted for the V0 population, and 169 to 257 cells were counted for the V1 population, according to brachial, thoracic or lumbar levels. Histograms are normalized according to the total amount of cells in each ventral IN population, which corresponds to 100%. (n = 3). (TIF) [file pone.0070325.s006.tif]
